# Downregulation of Enteroendocrine Genes Predicts Survival in Colon Cancer: A Bioinformatics-Based Analysis

**DOI:** 10.3390/ijms262211127

**Published:** 2025-11-18

**Authors:** Eloisa Martins da Silva, Marcella Cipelli, Mariana Aamaral do Amaral, Alvaro Pacheco-Silva, Niels O. S. Câmara, Vinicius Andrade-Oliveira

**Affiliations:** 1 Department of Nephrology, Paulista School of Medicine, Federal University of São Paulo, Pedro de Toledo Street, 669, Vila Clementino, São Paulo 04039-032, Brazil; apacheco@unifesp.br (A.P.-S.); niels@icb.usp.br (N.O.S.C.); 2 Department of Immunology, Institute of Biomedical Sciences, University of São Paulo, Av. Professor Lineu Prestes 1730, ICB IV, Butantã, São Paulo 05508-000, Brazil; marcella.cipelli@usp.br (M.C.); marianaamaral1231@gmail.com (M.A.d.A.); 3 Center for Natural and Human Sciences, Federal University of ABC, Avenida dos Estados, 5001, Santa Terezinha, Santo André 09210-580, Brazil

**Keywords:** colorectal cancer, intestinal epithelial cells, Paneth cells, enteroendocrine cells, bioinformatic, gene expression

## Abstract

Colorectal cancer (CRC) is the fourth most common and the third mostly deadly cancer globally. Even with alternative therapies, some patients do not respond to treatment. Identifying modulations in the tumor microenvironment (TME) of CRC is a significant challenge due to the complex and dynamic nature of the TME. The intestinal epithelium comprises different types of secretory lineage cells, including goblet, tuft, Paneth, and enteroendocrine cells (EECs). Yet the relevance of each subtype of secretory intestinal epithelial cell (IEC) within the TME is still debated. This study investigated the involvement of IECs in CRC development through an integrative bioinformatics analysis. We used publicly available datasets from the National Center for Biotechnology Information, the Cancer Genome Atlas Program, and the National Cancer Institute’s Proteomics Tumor Analysis Consortium, encompassing both human and mouse CRC samples. Our findings reveal a CRC microenvironment characterized by elevated expression levels of genes associated with WNT pathway activity. Remarkably, there was increased expression of Paneth cell-associated markers and transcription factors, such as *WISP1*, *LYZ*, *SOX9*, and *DEFA1*. Conversely, EEC-specific gene markers, such as *GCG* (encoding glucagon-like peptide-1) and *CHGA* exhibited significant downregulation in CRC tissue compared with healthy tissue, partially due to Paneth cell activity. Gene ontology analysis showed species-conserved downregulation in hormone/peptide secretion-related pathways in both mouse and human CRC. Of note, lower levels of GCG and CHGA correlated with reduced overall survival and demonstrated a correlation with the cell cycle, apoptosis, and proliferation. These results suggest that the disruption of enteroendocrine cell signaling is a hallmark of CRC development and may hold prognostic and therapeutic value in treating CRC patients.

## 1. Introduction

According to data from GloboCAN, 20 million new cancer cases occurred worldwide in 2022, resulting in a mortality rate of 9.7 million people. Colorectal cancer (CRC) is the third most diagnosed malignancy globally, surpassed only by sex-specific cancers (prostate cancer in men and breast cancer in women). This makes it the leading non-sex-specific cancer in terms of global incidence [1]. Several factors—including environmental, genetic, and inflammatory factors—are associated with CRC progression [2]. Furthermore, diet and obesity are linked to the development and progression of type II diabetes [3], which is, in turn, associated with the development of certain cancers, like CRC [4] and colitis-associated colorectal cancer (CAC). This suggests that the growth rate of the disease is directly linked to dietary habits [1,5,6]. Despite existing therapeutic alternatives (surgery, chemotherapy, radiation therapy, and immunotherapy), some patients do not respond to treatment. Additionally, modern lifestyle habits are recognized as risk factors for the development and progression of the disease. Importantly, the increasingly frequent appearance of early-onset CRC in young people has led to its ranking as the second most deadly cancer worldwide, significantly impacting the overall mortality rate [7]. This highlights the urgent need for further studies to unravel new molecules and pathways associated with CRC onset or progression that could be targeted for the development of new drugs and therapies.

CRC development is a complex process involving various molecules, pathways, cells, and environmental factors. It is directly linked to mutations in intestinal epithelial cells (IECs) and the breakdown of the epithelial barrier. The intestinal epithelium is composed of cell subtypes originating from intestinal stem cells (leucine-rich repeat-containing G-protein coupled receptor 5, LGR5+). Upon the induction of signaling pathways, including Wnt, β-catenin, and Notch, these stem cells coordinately activate specific transcription factors, promoting their differentiation into various IEC subtypes. IECs differentiate into either absorptive cells, known as enterocytes (differentiated by the transcription factor of the hes family bHLH transcription factor 1, HES1), or secretory cells, which include enteroendocrine cells (EECs), Tuft cells, goblet cells, and Paneth cells [8,9,10]. These secretory subsets are differentiated by the transcription factors Neurogenin 3 (NEUROG3), POU class 2 homeobox 3 (POU2F3), Kruppel-like factor 4 (KLF4), and SRY-box transcription factor 9 (SOX9), respectively.

In addition to their specific transcription factors, these secretory IECs are also characterized by the molecules or compounds that they produce and secrete. Tuft cells function as chemoreceptors, facilitating communication between the intestinal lumen, immune cells, and the neural network. They produce several molecules, such as neurotransmitters (acetylcholine), eicosanoids, and cytokines, (e.g., interleukin-25) [11]. Goblet cells primarily protect the epithelium by producing and secreting mucus, which acts as a protective barrier against pathogens [12]. Paneth cells are typically found in the small intestine and are uncommon in the large intestine under homeostasis. However, their presence in high numbers in the large intestine is an abnormal characteristic, often associated with metaplasia in patients with inflammatory bowel disease [13]. Paneth cells are responsible for producing essential antimicrobial proteins and peptides, such as alpha-defensin, playing a fundamental role in fighting infection and regulating the gut microbiome [14]. The last group of secretory lineage IECs is EECs. EECs represent about 1% of the epithelium, distributed throughout the intestinal epithelium, and are characterized by hormone production, found mainly in the small intestine and, to a lesser extent, in the large intestine. Hormone production is stimulated by diverse compounds, including macronutrients (glucose, amino acids, and fatty acids) and structural components of microorganisms, such as lipopolysaccharides [15]. Among the hormones produced by EECs, the group of incretins stands out—gastric inhibitory polypeptide (GIP) and glucagon-like peptide-1 (GLP-1). They are responsible for systemic functions, such as stimulating insulin secretion, controlling appetite in the central nervous system, and modulating inflammation and the immune system [16]. EECs and their peptide hormones regulate key functions of the gut epithelium, including digestion control, appetite regulation, glucose homeostasis, and immune function. This makes them potential targets for study in intestinal pathologies like CRC, given the role of hormones in mediating intestinal homeostasis [17,18].

It is known that IECs alter their transcriptional profiles and functions in response to the microenvironment, which can be modulated by various perturbations, such as those occurring in neoplastic conditions. However, it remains unknown whether secretory IECs, or their secreted products, are associated with the initiation or progression of CRC. We hypothesize that transcriptional and proteomic alterations affecting EECs and their hormones may destabilize intestinal epithelial homeostasis. To investigate this hypothesis, we performed an integrative analysis combining large-scale transcriptomics and proteomics data from human and experimental CRC samples. Using public gene expression databases, we assessed the abundance and enrichment of pathways related to markers of secretory IECs, with a specific focus on EECs and their hormones. Additionally, proteomics data were analyzed to investigate the relationship between EEC activity and the involved biological pathways. An EEC score was calculated, and its correlation with the proteomic landscape was explored through enrichment analysis, revealing pathways associated with cell proliferation, apoptosis, immune regulation, and inflammation.

Finally, to explore potential regulatory interactions, a mediation analysis was performed to investigate whether WNT pathway activation modulates EEC markers through Paneth cell activity. We aimed not only to characterize the abundance of specific markers of these populations compared to healthy tissue but also to identify potential prognostic markers associated with survival and response to chemotherapy in CRC patients. Taken together, these complementary approaches have allowed us to identify transcriptional and proteomic patterns that connect EEC function to tumor biology and clinical outcomes in CRC.

## 2. Results

### 2.1. Transcriptional Profile Analysis Reveals the Conserved Downregulation of EEC Markers in CRC

To gain a clearer understanding of the role that enteroendocrine hormones and secretory IECs play in the complex setting of CRC, we began by examining gene expression to identify potential molecular targets. We explored the presence or absence of secretory IEC markers by analyzing three different mouse CRC microarray datasets from the National Center for Biotechnology Information (NCBI) Gene Expression Omnibus (GEO) database (GSE86299, GSE31105, and GSE64658). CRC in these models was induced using a well-established inflammation-associated CRC model: azoxymethane (AOM) followed by the administration of dextran sulfate sodium (DSS) [19].

**Figure 1:**
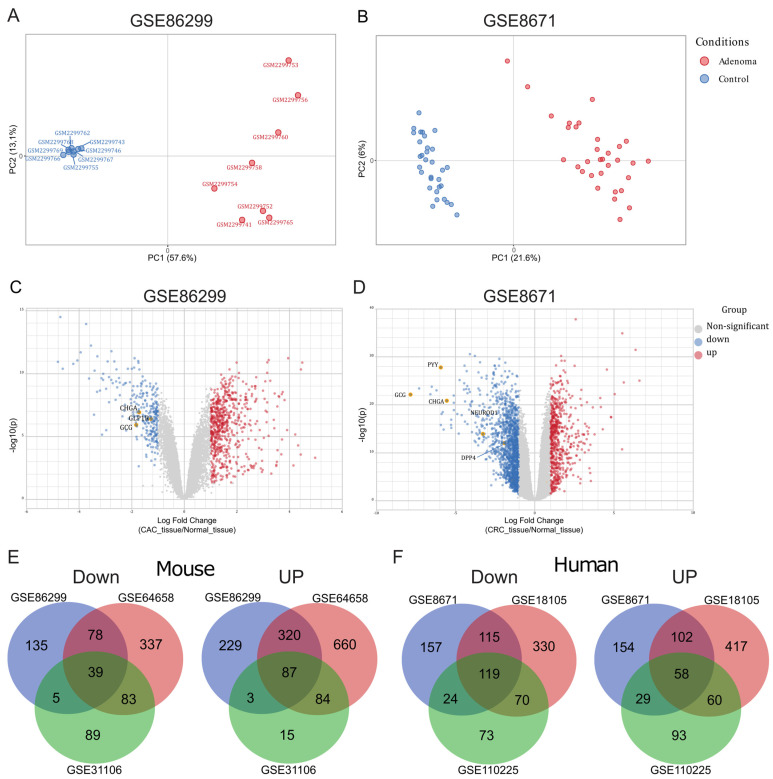
Differential expression analysis of microarray data from experimental and human colorectal cancer tissue. (**A**,**B**) Principal component analysis (PCA) plots of public microarray datasets from mouse GSE86299 (**A**) and human GSE8671 (**B**) tissue, comparing normal colon (blue) and colorectal cancer (CRC) (red). Mouse n=8 per group; human n=32 per group. These are representative datasets of a total of three analyzed (Figure S1). (**C**,**D**) Volcano plots displaying differential gene expression comparing murine (**C**) and human (**D**) CRC tissue with adjacent normal tissue. Genes down- and upregulated in cancer tissue are highlighted in blue and red, respectively. (**E**) Venn diagram illustrating the most common up- and downregulated genes across three experimental mouse models of colitis-associated colorectal cancer (CAC) derived from Gene Expression Omnibus (GEO) datasets. (**F**) Venn diagram illustrating the most common up- and downregulated genes across three human CRC datasets from GEO. The analysis was performed using the limma package version 3.16.2 with cut-offs of false discovery rate (FDR) <0.05 (Benjamini–Hochberg method) and $\log_{2}FC$ (fold change) <−1 and >1.

To assess data consistency and explore transcriptional variability, we conducted a principal component analysis (PCA). In mice, PCA clearly separated adenoma samples (red) from control samples (blue) (Figure 1A and Figure S1A,B). We then asked if human CRC data would show the same pattern. Applying the same method to three different human CRC datasets (GSE8671, GSE18105, and GSE110225) again showed a clear split between adenoma and nearby non-tumorous tissue (Figure 1B and Figure S1C,D).

Next, we carried out differential gene expression analysis using a false discovery rate (FDR) threshold of <0.05. Genes with a ${log}_{2}$ fold change ($\log_{2}FC$) ≤−1 were classified as downregulated (blue), and those with $\log_{2}FC\geq 1$ were classified as upregulated (red). In the mouse datasets GSE8671, GSE18105, and GSE110225, we found 257, 537, and 216 genes downregulated and 639, 1151, and 189 genes upregulated, respectively (Figure 1C and Figure S1E,F). As expected, genes related to inflammatory processes (such as chemokines and receptors, cell migration regulators, and growth factors) were upregulated. Strikingly, several key EEC genes—including chromogranin A (*Chga*), glucagon (*Gcg*), neurogenic differentiation 1 (*Neurod1*), peptide YY (*Pyy*), and glucagon-like peptide-1 receptor (*Glp1r*)—were consistently reduced in the AOM/DSS-induced CRC model.

The human CRC biopsy datasets revealed a similar pattern, with 634, 415, and 286 genes downregulated and 637, 343, and 240 genes upregulated, respectively. As in mice, genes involved in EEC differentiation and hormone production were consistently decreased in tumor tissue compared with normal tissue (Figure 1D and Figure S1G,H). Taken together, these results show distinct transcriptional profiles between adenoma and normal tissue in both mouse and human CRC, with a consistent decrease in the expression of EEC-associated genes in tumors.

### 2.2. Antimicrobial Peptide-Related Pathways Are Upregulated While Hormonal Processes Are Downregulated in Colorectal Cancer

After identifying differences in gene expression, we examined which biological pathways were most affected in tumor samples. To analyze unbiased pathways, we first used a Venn diagram to identify genes consistently upregulated or downregulated across all three mouse datasets and all three human datasets. In mice, 39 downregulated genes (Figure 1E, left) and 87 upregulated genes (Figure 1E, right) were common to all studies. In humans, 119 downregulated genes (Figure 1F, left) and 58 upregulated genes (Figure 1F, right) were shared among the datasets.

**Figure 2:**
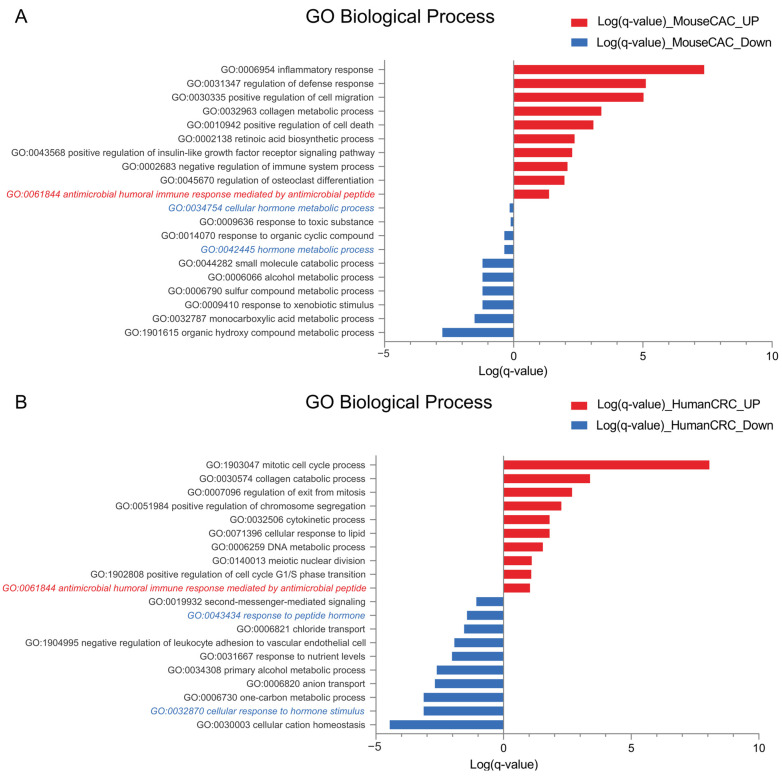
Antimicrobial peptide-related pathways are upregulated while hormonal processes are downregulated in colorectal cancer. (**A**) GO biological process for up- (red) and downregulated (blue) pathways based on common differentially expressed genes from Venn diagrams in mouse colitis-associated colorectal cancer (CAC) studies. (**B**) GO biological process for up- (red) and downregulated (blue) pathways based on common differentially expressed genes from Venn diagrams in human colorectal cancer (CRC) studies. Biological processes related to Paneth cells and enteroendocrine cells are highlighted. Analysis was performed using the Metascape platform.

We then performed Gene Ontology (GO) biological process enrichment analysis on these shared genes. In mice, 295 pathways were enriched in the upregulated gene set, while 66 pathways were associated with the downregulated set (Tables S1 and S2). In humans, we found 157 and 167 pathways associated with up- and downregulated genes, respectively (Tables S3 and S4). As expected, based on existing data [20], for the upregulated genes in the tumor context, the increased pathways were mainly related to the inflammatory response and positive regulation of the cell cycle (Figure 2A,B).

One notable and species-conserved finding was the enrichment of pathways linked to antimicrobial peptide-mediated immune responses among the upregulated genes in tumor samples (Figure 2A,B). Paneth cells, known producers of antimicrobial peptides, interact with the gut microbiota and can influence immune cell activity [21]. In contrast, pathways associated with hormonal processes—many of which depend on products secreted by EECs—were consistently enriched in the downregulated gene set (blue) in tumor tissue. These analyses suggest that tumor samples exhibit conserved stronger activity in antimicrobial peptide-related pathways, potentially driven by Paneth cells, and weaker activity in hormone-related functions of EECs.

### 2.3. Upregulation of Paneth Cell Markers and Downregulation of EEC Genes Is Conserved Across Colon Tumor Origin

**Figure 3:**
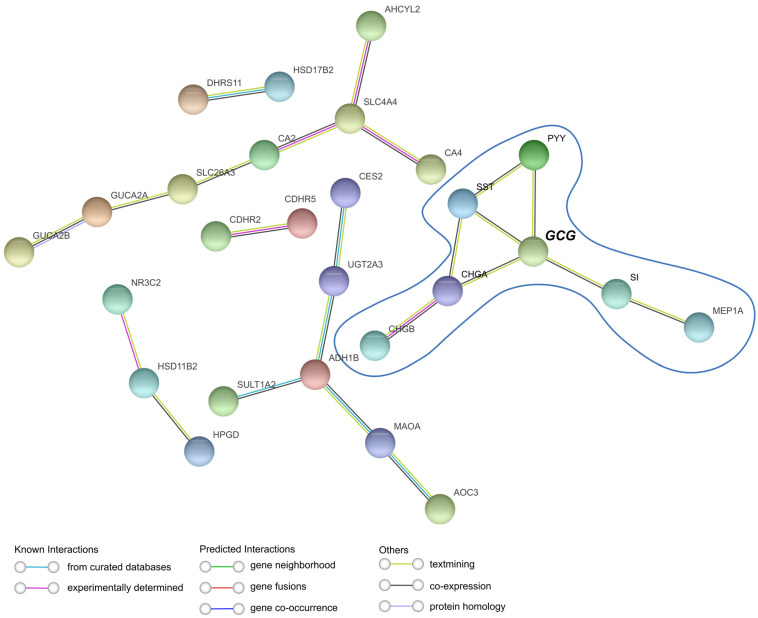
Upregulation of Paneth cell-related markers and downregulation of enteroendocrine cell genes in experimental and human colorectal cancer. Differential gene expression between tumor and normal samples from humans (GSE86299, GSE18105, and GSE110225), represented by a circle, and mice (GSE8671, GSE31106, and GSE64658), represented by a square. The *y*-axis lists genes selected as specific markers of secretory intestinal epithelial cells, along with their transcriptional regulators, as previously described in the literature. The *x*-axis represents the $-\log_{10}\left(\mathrm{adj}.p\right)$. The vertical dotted line indicates the cut-off for FDR <0.05. The color intensity denotes the direction and level of the difference $\log_{2}FC$ between tumor and normal tissue, while the size of the bubble is related to the level of significance of the $\mathrm{FDR}-\log_{10}\left(\mathrm{adj}.p\right)$. Bubble-based heatmap generated using R (version 4.x; R Core Team, 2024) with the ggplot2 (version 4.2), readr (version 2.1.5), dplyr (version 1.1.4), and scales (version 1.4.0) packages.

Given the observed pathway alterations, we explored whether markers and transcription factors associated with other secretory IEC subtypes were also dysregulated. To address this, we examined marker genes for secretory IEC subtypes, including critical differentiation transcription factors and their secreted products (Table 1). For a marker to be considered significant, it had to be differentially expressed in the same direction (up- or downregulated) across all datasets. We generated a heatmap/bubble plot using the markers listed in Table 1, where the bubble size represents the adjusted *p*-value ($-\log_{10}\left(\mathrm{adj}.p\right)$) and the color intensity indicates the $\log_{2}FC$ (red for ≥2 and blue for ≤−2). Then, we selected tuft cell markers, namely SRY-box transcription factor 4 (*SOX4*) and POU2F3, which did not exhibit a consistent pattern of regulation (Figure 3). In contrast, goblet cell markers, such as *KLF4*, mucin 2 (*MUC2*), and SAM pointed domain-containing Ets transcription factor (*SPDEF*), were consistently downregulated in tumor tissue, aligning with the literature describing a reduced goblet cell population in the neoplastic intestinal epithelium [22,23].

Consistent with our enrichment analysis, transcription factors essential for EEC differentiation—such as *NEUROD1* and Aristaless-related homeobox (*ARX*)—were downregulated in both the murine and human tumor microenvironments (TMEs). Canonical EEC markers and hormone genes (e.g., *GCG* and *PYY*) were likewise reduced in tumor tissue (Figure 3). In contrast, Paneth cell markers, including defensin alpha 1 (*DEFA1*), lysozyme (*LYZ*), and *SOX9*, were upregulated in tumor tissue. Additionally, WNT1-inducible signaling pathway protein 1 (*WISP1*)—a transitional marker of Paneth cell differentiation [10]—also showed increased expression in tumor tissue (Figure 3). Together, these data suggest a shift in IEC marker expression in tumor samples, with an increase in Paneth cell markers and a decrease in EEC and goblet cell markers.

We next explored whether this shift also occurs in spontaneously developing CRC, without inflammation induction, by examining the dataset GSE107139, obtained from the APC^min^ CRC model [42]. Consistent with our observations, APC^min^ animals exhibited the downregulation of EEC markers and upregulation of Paneth cell markers in tumor tissue (Figure S2A,C). We then assessed whether human CAC exhibits a similar pattern using the GSE37283 dataset. This dataset confirmed the upregulation of *LYZ* and *WISP1*, alongside the downregulation of EEC identity genes (*GCG*, *PYY*, *CHGA*, and *NEUROD1*) (Figure S2B,C). Interestingly, goblet cell markers were not reduced in this CAC dataset, suggesting that this phenomenon may be specific to CAC, further reinforcing the centrality of Paneth cell expansion and EEC loss as conserved features across tumorigenesis (Figure S2A–C).

**Figure 4:**
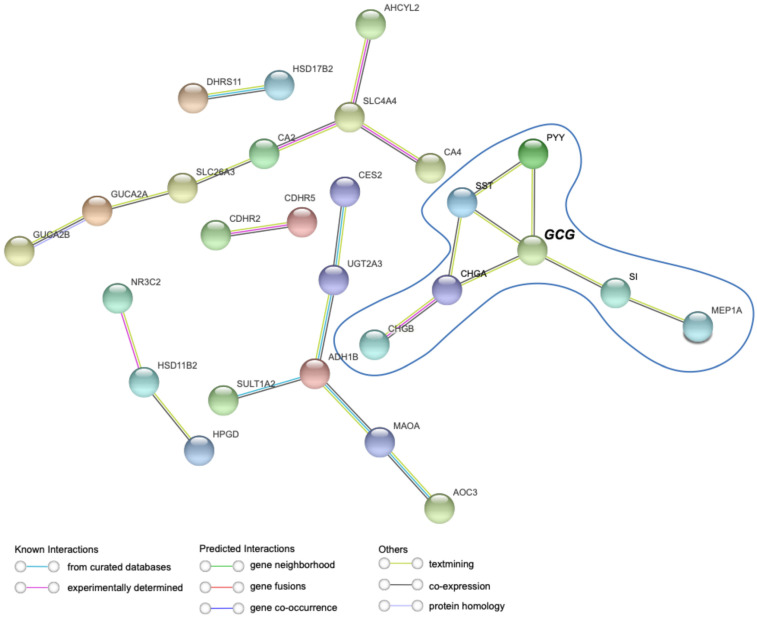
The STRING interaction shows *GCG* as the gene with the highest number of interactions with other enteroendocrine cell markers. STRING analysis of common downregulated genes obtained from Venn diagrams in human colorectal cancer (CRC) using the minimum required interaction score with high confidence. Analysis performed using the string-db platform.

The STRING platform for protein–protein interaction (PPI) network analysis reveals the largest interaction cluster among the downregulated genes (Figure 1F), mainly involved enteroendocrine-related genes, with *GCG* as the central node (Figure 4). This is in line with the enrichment analysis highlighting downregulated hormonal processes (Figure 2B), suggesting a key role for EEC genes in human CRC. However, upregulated genes did not form a highly connected network related to Paneth cells, with interactions restricted to only two genes (Figure S4A).

Together with the gene expression data, these results suggest a balance favoring a decrease in EECs and an increase in/maintenance of Paneth cell markers in the TME, with the GCG gene emerging as a potential marker of this phenomenon.

### 2.4. RNA-Seq Analysis from TCGA Confirms That EEC Markers Are Among Most Downregulated Genes

**Figure 5:**
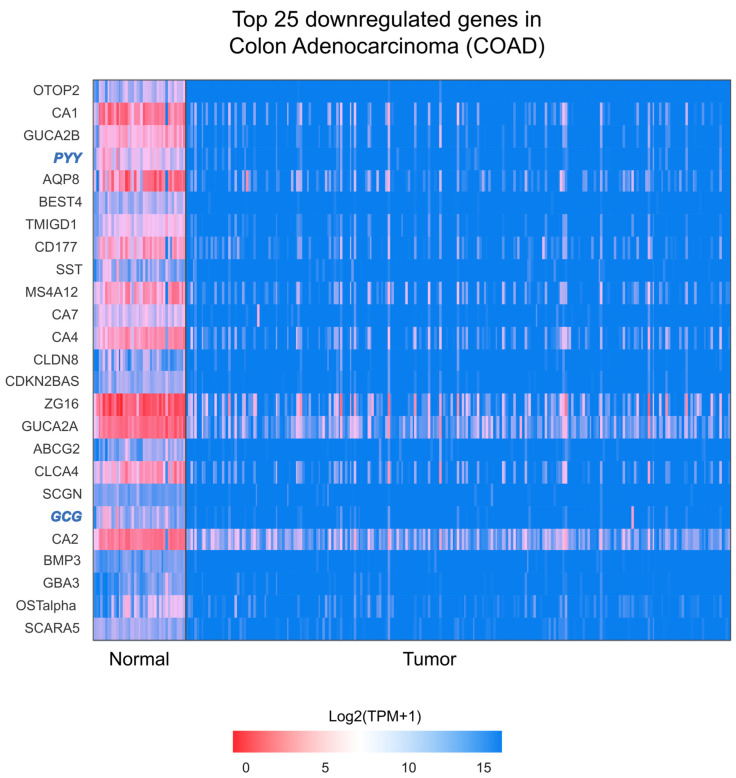
RNA-Seq analysis from the Cancer Genome Atlas (TCGA) platform identified glucagon (*GCG*) and peptide YY (*PYY*) as two of the top 25 most downregulated genes in colorectal adenocarcinoma tumor samples. The top 25 downregulated genes in human colon adenocarcinoma (COAD) tissue from the TCGA RNA-Seq database. The color range reflects the ${log}_{2}$-normalized transcript per million (${log}_{2}TPM+1$) values comparing normal and colorectal cancer tissue. The *y*-axis represents the top 25 genes (symbol), while the *x*-axis represents the groups (normal and tumor). Among our target genes, *GCG* and *PYY* were found, which are related to enteroendocrine cells and are highlighted in blue. Analysis performed using the UALCAN platform.

Given the consistent shift observed, we used the Cancer Genome Atlas Program (TCGA) database, which contains RNA sequencing data from patients with colon adenocarcinoma (COAD), to identify potential therapeutic targets. We validated the downregulation of EEC markers and others using a separate COAD cohort. Remarkably, two EEC markers—*GCG* and *PYY*—were among the top 25 most downregulated genes in COAD tissue (Figure 5). In contrast, none of the top 25 upregulated genes were associated with other IEC subtypes (Figure S5A). This indicates that EEC-related genes are disproportionately represented among the downregulated transcripts, reinforcing the observation of consistent EEC marker downregulation across COAD tumor samples.

### 2.5. Modulation of EEC and Paneth Cell Markers Occurs Independently of Tumor Stage

**Figure 6:**
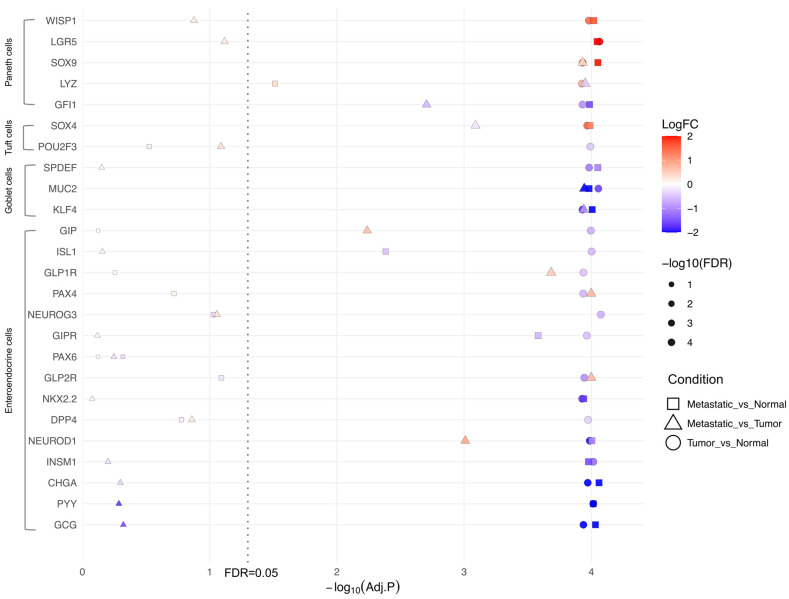
RNA-Seq analysis from the Cancer Genome Atlas (TCGA) colon adenocarcinoma (COAD) database showing the differential expression of intestinal epithelial cell (IEC) markers in metastatic colorectal cancer. Differential expression comparison from the TCGA RNA-Seq of COAD between tumor and normal (circle), metastatic and normal (square), and metastatic and tumor (triangle). The *y*-axis lists genes that were selected as previously described. The *x*-axis represents the $-\log_{10}\left(\mathrm{adj}.p\right)$. The vertical dotted line indicates the cut-off for FDR<0.05. The color intensity denotes the direction and level of the difference ($\log_{2}FC$) between tumor and normal tissue, while the size of the bubble is related to the level of significance of the FDR ($-\log_{10}\left(\mathrm{adj}.p\right))$. Bubble-based heatmap generated using R (version 4.5.1; R Core Team, 2024) with the ggplot2 (version 4.0.0), readr (version 2.1.5), dplyr (version 1.1.4), and scales (version 1.4.0) packages.

We investigated whether this IEC marker shift is related to tumor development, which is staged based on the extent of infiltration and metastasis [43]. When comparing COAD stages (I through IV), the expression of most IEC-associated genes did not differ significantly across stages (Figure S6A–X). Nonetheless, an analysis of metastatic versus non-metastatic COAD tissue revealed that EEC-related genes (*GCG*, *PYY*, *CHGA*, *NEUROD1*) were consistently downregulated in both tumor and normal tissue and between metastatic and normal tissue (Figure 6). This suggests that the reduction in EEC markers occurs in the early stages of tumorigenesis and persists throughout tumor development, even in metastatic progression. Paneth cell markers showed an increase in tumor samples regardless of the tumor stage (Figure 6).

### 2.6. Protein-Level Evidence Supports EEC/Paneth Cell Marker Balance in CRC

To confirm the mRNA findings at the protein level, we utilized the Human Protein Atlas. As expected, 100% of all normal tissue was positive for EEC markers (GCG, PYY, CHGA, and NK2 homeobox 2 (NKX2.2)) and nearly 100% for Paneth markers (DEFA1, LYZ, SOX9, and WISP1) (Figure S3A). While the percentage of Paneth cell markers remained unchanged in CRC and normal tissue, CRC tissue positive for EEC markers was rare (Figure S3A). Immunohistochemistry imaging visualized the loss of the ECC marker CHGA and the preservation of the Paneth cell marker DEFA1 (Figure S3A–C).

**Figure 7:**
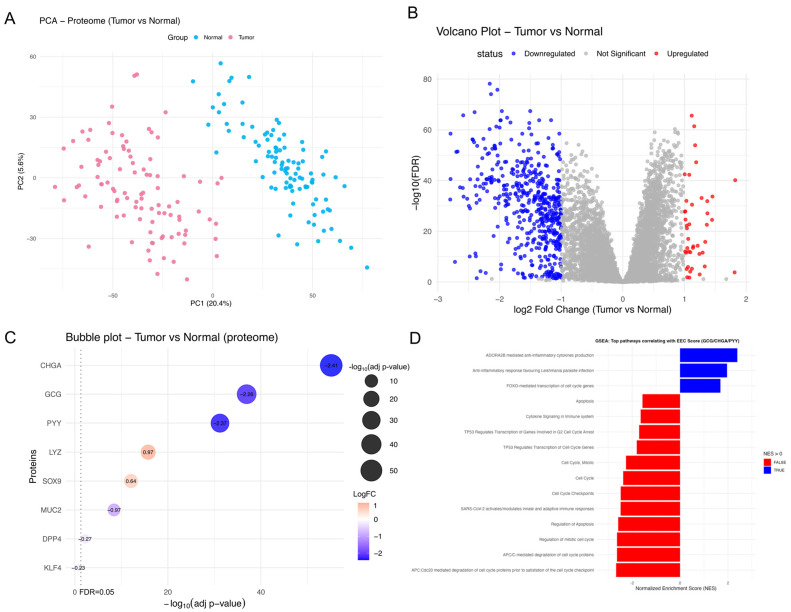
Protein-level analysis reveals a balance between enteroendocrine cell (EEC) and Paneth cell markers in CRC. (**A**–**D**) Proteomics dataset from the CPTAC database (PDC000116), comparing tumor and normal samples. Adjusted p-values $\left(\mathrm{FDR}\right)$ were calculated using the Benjamini–Hochberg method implemented in limma. (**A**) PCA plot of normal (blue) and tumor (red) human samples from proteomics data. (**B**) Volcano plot considering the cut-offs mentioned above. $\log_{2}FC$ protein expression values downregulated (blue) <−1 and upregulated (red) >1 are highlighted. (**C**) Bubble-based heatmap using proteomics data for intestinal epithelial cell markers. The *y*-axis lists genes that were selected as previously described. The *x*-axis represents the $-\log_{10}\left(\mathrm{adj}.p\right)$. The vertical dotted line indicates the cut-off for FDR<0.05. The color intensity denotes the direction and level of the difference $\log_{2}FC$ between tumor and normal tissue, while the size of the bubble is related to the level of significance of the FDR $\left(-\log_{10}\left(p.\mathrm{adj}\right)\right).$ The number inside each bubble refers to the $\log_{2}FC$. (**D**) Bar graph showing the main Reactome pathways correlated with the EEC score (calculated from the mean of the GCG, CHGA, and PYY proteins). Pathways in blue, where normalized enrichment score $\left(\mathrm{NES}\right)$ >0, represent processes positively associated with an increase in the EEC score, while those in red, $\left(\mathrm{NES}\right)$ >0, are negatively associated. Analysis was performed using R (version 4.5.1) with the limma (version 3.64.3), fgsea (version 1.34.2), clusterProfiler (version 4.16.0), reactome.db (version 1.92.0), org.Hs.eg.db (version 3.21.0), mediation (version 4.5.1), ppcor (version 1.1) and tidyverse (version 2.0.0) packages.

We then analyzed the National Cancer Institute’s Proteomics Tumor Analysis Consortium (CPTAC) dataset to see if the transcriptional profile was maintained at the protein level. Using the PCA plot, we verified the sample quality and group separation (normal tissue (blue) and tumor tissue (red)) (Figure 7A). Differential expression analysis, using the same cut-offs (FDR<0.05; $\log_{2}FC<-1>\mathrm{and}>1$), identified eight relevant proteins: CHGA, GCG, PYY, LYZ, SOX9, MUC2, DPP4, and KLF4 (Figure 7B). This analysis confirmed the mRNA findings: a reduction in GCG, CHGA, and PYY was associated with an increase in LYZ and SOX9 (Figure 7C), supporting a balance between these two cell populations in the TME.

### 2.7. EEC Modulation in TME Is Linked to Apoptosis and Proliferation and Driven by WNT Signaling

WNT activation is known to cause an increase in Paneth cells [44]. Nevertheless, the mechanism underlying the decrease in EECs within the TME remains unclear. To investigate this, we performed a mediation analysis using the CPTAC proteomics dataset. This analysis tested whether the effect of WNT activation on EEC marker expression is mediated indirectly through Paneth cell expansion or directly. Our findings showed that the wnt_score (calculated based on the expression of CTNNB1, MYC, AXIN2) had a direct negative effect on EEC proteins (GCG, CHGA, and PYY), as shown by the average direct effect (ADE) index (Table 2). This indicates that increased Wnt activity is directly associated with reduced EEC marker expression. While the average causal mediation effect (ACME) index showed a trend of a negative indirect effect via SOX9 and LYZ, it was not statistically significant (ACME <0.05), suggesting that the Wnt signaling modulation of EEC markers occurs predominantly through a direct mechanism (Table 2). Nonetheless, mediation through LYZ showed a trend toward significance, pointing to a possible partial contribution of this protein expression to the Wnt–EEC regulatory axis.

To correlate EEC downregulation with potential mechanisms, we performed a complementary functional enrichment analysis. We calculated an EEC score for each sample based on the mean normalized abundance of the GCG, CHGA, and PYY proteins and correlated it with all quantified proteins for gene set enrichment analysis (GSEA). The analysis revealed that samples with lower EEC scores showed enrichment in proliferative and apoptotic pathways such as “cell cycle” and “TP53 transcriptional regulation” (Figure 7D). Together, these findings suggest that reduced EEC activity in CRC correlates with the activation of cell cycle and apoptosis pathways, potentially related to the loss or differentiation of EECs.

### 2.8. High GCG and CHGA Expression Correlates with Improved Overall Survival

**Figure 8:**
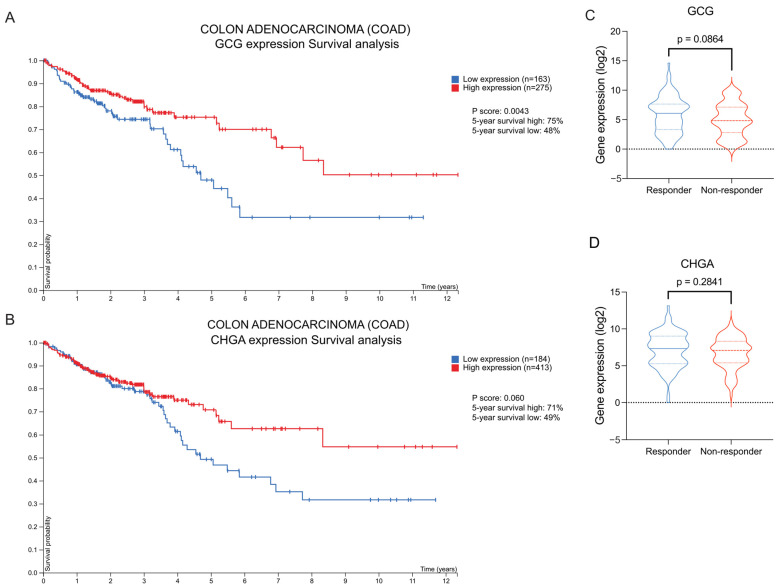
Low expression levels of enteroendocrine genes correlate with worse clinical outcomes in patients with colon adenocarcinoma (COAD) tumors. (**A**,**B**) Five-year survival curves of patients with COAD, stratified based on GCG (**A**) and CHGA (**B**) expression. Kaplan–Meier plot obtained from Protein Atlas. (**C**,**D**) Bar graph analysis of the gene expression levels of GCG (**C**) and CHGA (**D**) in COAD tumor patients classified as responders or non-responders to chemotherapy. The values are expressed as ${log}_{2}$-normalized. Data obtained from rocplot.com.

We evaluated whether the Paneth cell marker upregulation and EEC marker downregulation could predict patient survival and treatment responses. A survival analysis using the Human Protein Atlas database was performed to evaluate the prognostic relevance of EEC and Paneth cell markers in CRC patients. Patients were stratified into high- and low-expression groups based on transcript levels, and overall survival was assessed using Kaplan–Meier estimates and the log-rank test. Interestingly, only GCG and LYZ demonstrated a significant association with patient outcomes (Figure 8A,B; Table S5). Specifically, high GCG expression was associated with longer 5-year survival in COAD patients (Figure 8A). Similarly, the EEC marker CHGA showed a positive association between elevated expression and prolonged survival (Figure 8B). None of the Paneth cell markers were significantly associated with 5-year patient survival (Table S5). We then investigated whether the expression of these genes correlated with chemotherapy responsiveness. Our analysis showed that chemotherapy-responsive patients tended to have higher *GCG*, while there was no difference regarding *CHGA* expression (Figure 8C,D). Collectively, these findings indicate that the downregulation of EEC markers directly impacts patient survival and, in the case of GCG, may also be linked to the chemotherapy response.

## 3. Discussion

In silico analyses using publicly available sequencing data are a powerful tool for deriving comprehensive overviews and translational conclusions about biological phenomena. Our findings reveal that tumor tissue from CRC patients and experimental models exhibits consistent differences in IEC markers when compared to non-neoplastic tissue. This reinforces the concept that distinct IEC subtypes play key but contrasting roles in the TME. Among the secretory IECs, goblet cells—responsible for mucus production—are particularly affected during tumor development. Their reduced numbers in the tumor context, associated with decreased mucus secretion, are linked to a worse prognosis and more aggressive disease [45]. Our work agrees with the established literature, showing transcriptional alterations and the downregulation of critical differentiation and function genes for goblet cells (*MUC2*, *KLF4*, and *SPDEF*). On the other hand, tuft cells play a dual role in the tumor microenvironment depending on the context. They can promote tumor progression by promoting tumor growth and metastasis through epithelial–mesenchymal transition [46,47,48]. In our analyses, however, the markers of these cells showed heterogeneous expression patterns, suggesting the necessity of additional analyses focusing on other markers and signaling pathways related to this cell subtype.

The role of EECs in tumor biology is controversial, with studies suggesting both promoting and protective influences in different cancers [49,50]. Previous CRC reports, which primarily focused on single EEC markers (e.g., CHGA or PYY), have yielded inconsistent prognostic results [51,52,53,54]. Conversely, NEUROD1 has been implicated in promoting tumor growth and metabolic reprogramming in CRC [55,56]. This duality likely reflects the specificity of the tumor type, the complexity of the hormones’ systemic effects, and their modulation of metabolic and immunological pathways.

Our study advances beyond previous work by providing the comprehensive, cross-species, transcriptome- and proteome-wide characterization of EEC marker expression in CRC. Instead of isolated proteins, we identified a downregulated network of EEC markers (CHGA, GCG, PYY, NEUROD1) associated with tumor processes, reduced survival, and poor chemotherapy responses. This reveals that EEC loss is not merely a histological feature but is part of the functional reprogramming of the intestinal TME, paving the way for mechanistic studies on the EEC–CRC relationship.

While the exact role of EECs in CRC remains unclear, the hormones that they produce may influence neoplastic processes, particularly locally in the intestine. The incretin group (GIP and GLP-1) is of high interest. A recent study reported that patients treated with incretin-based antidiabetic drugs exhibited a lower risk of developing CRC compared with those receiving other medications, independently of weight loss [57]. Since GCG encodes the precursor of GLP-1, the observed reduction in GCG and other key EEC markers (CHGA, NEUROD1, PYY) in tumor tissue, combined with these external clinical data, suggests that EECs and incretin hormones may exert an anti-tumor role in colorectal carcinogenesis.

Interestingly, Paneth cells are producers of antimicrobial peptides that interact with the gut microbiota and modulate immune cells [21], showing a contrasting pattern, being increased in tumor tissue, as indicated by the upregulation of the genes *LYZ*, *DEFA1*, *SOX9*, and *WISP1*. This, combined with the downregulation of EEC and goblet cell markers, strengthens the hypothesis of a functional balance or shift between secretory IEC populations in the TME. Our GO enrichment analysis supports this shift: upregulated genes were consistently correlated with antimicrobial peptide-mediated immune processes, while downregulated genes were associated with hormonal processes, the peptide hormone response, and hormonal metabolic processes. It is important to note that Paneth cells are typically absent in the large intestine; therefore, the increase in their markers in CRC highlights the complexity of tumor cell development, potentially representing metaplasia. Further studies are warranted to understand the impact of this cellular shift on tumor progression, anti-tumor immunity, and changes in the microbiota composition [39,58].

The WNT pathway is essential for all secretory lineages [59], but WNT hyperactivation specifically promotes Paneth cell differentiation [44]. The upregulation of the WNT signaling marker *WISP1* in tumor tissue suggests that the TME upregulates Paneth cells through increased WNT signaling. This is consistent with the finding that Paneth cell accumulation in CRC is linked to WNT/β-catenin signaling activation and a poor prognosis [60]. Crucially, our results show that WNT signaling is directly associated with both an increase in the Paneth cell population and a decrease in EECs. Mediation analysis indicated that this WNT–EEC relationship is primarily direct, with only a limited, non-significant contribution from Paneth cells (LYZ showed a trend). This suggests that WNT hyperactivation in the TME promotes the secretory cell shift, favoring Paneth cells over EECs, via a direct mechanism, potentially affecting intestinal hormone secretion and local immune modulation. Our results point to a possible relevant functional impact of WNT signaling on the cellular composition in the TME. However, experiments are needed to confirm this mechanism and to determine which possible modulations in this pathway and these cell populations could influence tumor formation.

In the TME, several processes and pathways occur that are overactivated, including pathways related to apoptotic processes, DNA repair, p53 signaling, cell proliferation, and the cell cycle, which are characteristic of tumor development. We investigated the functional impact of EEC downregulation. Samples with lower EEC scores were associated with the activation of the cell cycle, apoptotic and proliferative pathways in GSEA. This indicates that reduced EEC activity correlates with greater proliferation and apoptosis susceptibility in the TME, supporting the notion that WNT-mediated EEC downregulation alters the cellular composition and impacts key tumor-related biological processes [61].

Our findings across multiple cohorts consistently demonstrate conserved, dynamic modulation between EECs and Paneth cells in the TME. This cellular interaction appears to have important clinical implications, as it links to the therapeutic response and patient outcomes. Here, we identified *GCG* and *CHGA* as key genes associated with survival in CRC patients. High expression of these EEC markers correlated with a better prognosis and significantly improved overall survival in COAD patients. This is consistent with a recent study reporting a positive correlation between GCG expression in immune cells within COAD tumors and favorable patient outcomes [62]. Future studies in patient cohorts will be needed to adjust for clinical variables and validate the findings.

In summary, the molecular signatures of epithelial subpopulations not only reflect tumor biology but may also serve as a prognostic indicator and potential therapeutic target in CRC. A limitation of our study, based exclusively on public datasets, is that some targeted markers were absent from the protein expression datasets, and, in some cases, the antibodies failed to produce detectable staining in any of the tissues examined. These highlight the critical need for follow-up experimental work and clinical approaches to validate these transcriptomic patterns and mechanistically confirm the importance of this WNT–Paneth–EEC balance in CRC development.

## 4. Materials and Methods

### 4.1. Database Collection

Datasets for large-scale gene expression profiling were retrieved from the GEO at NCBI, containing intestinal samples from both animal models of CRC and human subjects diagnosed with CAC. For subsequent analyses, we selected the three datasets available on the NCBI platform that had the same cancer induction model as well as the same sequencing and data analysis method. From mice, we included GSE86299, GSE31105, and GSE64658, representing experimental models of CAC induced by azoxymethane and dextran sulfate sodium, as well as GSE107139 from APC^min^ mice, which spontaneously develop colorectal tumors due to a mutation in the tumor suppressor gene APC. From human samples, we analyzed datasets derived from CRC biopsies (GSE8671, GSE18105, and GSE110225) and from cases of CAC (GSE37283).

### 4.2. Differential Expression Analysis Datasets from NCBI

Microarray differential expression was analyzed using the Network Analyst platform [63] in conjunction with the limma (version 3.16.2) R package. Statistical cut-offs were set at an FDR<0.05 (Benjamini–Hochberg method) and $\log_{2}FC<-1>\mathrm{and}>1$. PCA graph and volcano plots were generated based on these criteria. A bubble-based heatmap was created using R (version 4.5.1) with the ggplot2 (version 4.0.0), readr (version 2.1.5), dplyr (version 1.1.4), and scales (version 1.4.0) packages. Genes passing the established cut-offs were separated into up- and downregulated lists for subsequent enrichment analysis [64].

### 4.3. RNA-Seq Differential Expression Analysis from TCGA Database

RNA-Seq data from the TCGA-COAD cohort were analyzed for differential expression segmented by tumor stage (I, II, III, and IV, based on the American Cancer Society criteria) using the Oncodb platform [65], applying the same FDR<0.05 and $\log_{2}FC<-1>\mathrm{and}>1$ cut-offs as above. The top 25 up- and downregulated genes in COAD were obtained from the integrated cancer data analysis platform Ualcan [66]. PPI network analysis of differentially expressed genes was performed using the STRING platform [67].

### 4.4. Proteomics Analysis from CPTAC, Differential Expression, and GSEA

Differential protein expression analysis between tumor and normal samples was performed on the CPTAC database (PDC000116). Linear models were used, and adjusted *p*-values (FDR<0.05) were calculated using the Benjamini–Hochberg method implemented in limma. To investigate pathway-level associations, GSEA was conducted. The EEC score was calculated as the mean expression of EEC markers (GCG, CHGA, PYY). All proteins were ranked by Pearson correlation with the calculated EEC score. The enrichment of Reactome pathways was analyzed using fgseaMultilevel with 1000 bootstrap iterations, and adjusted *p*-values were corrected by FDR. All analyses were conducted in R (version 4.5.1) using the packages limma (version 3.64.3), fgsea (version 1.34.2), clusterProfiler (version 4.16.0), reactome.db (version 1.92.0), org.Hs.eg.db (version 3.21.0), mediation (version 4.5.1), ppcor (version 1.1), and tidyverse (version 2.0.0).

### 4.5. Mediator Analyses with Proteomics Data

Mediator analysis was performed using the CPTAC dataset (PDC000116) to examine the indirect effects of WNT pathway activity on EEC markers. The mediate() function was used to estimate the ACME and the ADE, with non-parametric bootstrapping (1000 simulations) to calculate confidence intervals and *p*-values. Expression data were log-transformed and standardized prior to modeling. The WNT pathway activation score (wnt_score) was calculated as the mean expression of canonical WNT target proteins (CTNNB1, MYC, AXIN2). First, the mediation model was examined, in which the expression of LYZ or SOX9 (Paneth cell markers) was regressed on the wnt_score (LYZ or SOX9~wnt_score). Second, the outcome model was examined, in which the expression of GCG, PYY, or CHGA (enteroendocrine marker) was regressed on either LYZ or SOX9 or the WNT score (GCG/PYY/CHGA~LYZ/SOX9 + wnt_score).

### 4.6. Survival Analysis and Chemotherapy Responder Correlation

Survival analysis and the association of gene/protein expression with patient outcomes were performed using the Human Protein Atlas platform for the TCGA COAD patient database [68]. Overall survival was evaluated using Kaplan–Meier estimates and assessed with the log-rank test. It is important to note that these analyses did not account for patient variables such as stage, age, or sex. Moreover, the correlation between chemotherapy responsiveness and the expression of GCG and CHGA expression was performed using the rocplot.com platform [69].

## 5. Conclusions

This integrative study, combining transcriptomics and proteomics analyses across human and experimental models, reveals a conserved and dynamic shift in the gene expression balance between EEC and Paneth cell markers within the CRC TME. This balance is accompanied by the upregulation of biological pathways associated with antimicrobial peptide-mediated immune responses and, conversely, the downregulation of hormone-related processes. Furthermore, we provide robust evidence linking the reduced expression of key enteroendocrine-associated genes—*GCG* and *CHGA*—to poorer survival outcomes in patients with CRC. Mediation analysis further suggests that WNT pathway activation may directly suppress enteroendocrine markers, as well as partly through Paneth cell activity. Finally, proteome-based enrichment analysis demonstrated that samples with lower EEC scores were enriched for cell cycle and apoptosis pathways, suggesting that the loss of EEC activity is tightly linked to key proliferative and cell death mechanisms characteristic of cancer progression. Taken together, these results establish that the disruption of EEC signaling and the simultaneous expansion of Paneth cell markers are hallmarks of CRC progression, holding significant prognostic and therapeutic value for the treatment of CRC patients.

## Figures and Tables

**Table 1:** Genes related to each intestinal epithelial cell subtype and their descriptions.

Cell Type	Gene	Gene Function/Description	Reference
Enteroendocrine cell	*GCG*	GLP-1/2 hormone L-cell marker	[24]
	*PYY*	Hormone secreted by L cells	
	*CHGA*	Classical marker	[15]
	*INSM1*	Neuroendocrine transcription factor	[25]
	*NEUROD1*	Neuroendocrine differentiation	[26]
	*DPP4*	Incretin degradation (GLP-1, GIP)	[27]
	*ARX*	Regulates the fate of endocrine subtypes	[28]
	*NKX2.2*	Required for intestinal endocrine differentiation	[24]
	*GLP2R*	GLP-2 receptor	[29]
	*PAX6*	Regulates EEC subtypes	[24]
	*GIPR*	GIP receptor	
	*NEUROG3*	EEC master regulator	[30]
	*PAX4*	Intestinal endocrine development	[24]
	*LMX1A*	Regulates serotonin	[31]
	*GLP1R*	GLP-1 receptor	[24]
	*ISL1*	Endocrine regulation	
	*GIP*	Hormone produced by K cells	
Goblet cell	*KLF4*	Goblet cell differentiation	[32]
	*MUC2*	Major secreted mucin	[33]
	*SPDEF*	Essential for goblet cells	[34]
Tuft cell	*POU2F3*	Tuft cell master regulator	[35]
	*SOX4*	Tuft development	[36]
Paneth cell	*GFI1*	Regulation of Paneth cells	[37]
	*DEFA1*	Antimicrobial peptide produced by Paneth cells	[38]
	*LYZ*	Lysozyme classic marker	[39]
	*SOX9*	Essential transcription factor	[10]
	*WISP1*	Maintains niche and Paneth differentiation	[40]
Stem cell	*LGR5*	Intestinal stem cell marker	[41]

**Table 2:** Mediation analysis of wnt_score effects on EEC marker expression through SOX9 and LYZ.

Mediator	Outcome	ACME_ Estimate	ACME_p	ADE_ Estimate	ADE_P Value	Summary
SOX9	CHGA	−0.39	0.27	−1.15	0.02	Direct effect of wnt_score on CHGA, independent of SOX9
SOX9	GCG	−0.10	0.82	−1.12	0.03	Direct effect of wnt_score on GCG, independent of SOX9
SOX9	PYY	−0.09	0.81	−1.37	0.01	Direct effect of wnt_score on PYY, independent of SOX9
LYZ	CHGA	−0.18	0.18	−1.36	0.0001	Direct effect of wnt_score on CHGA, independent of LYZ
LYZ	GCG	−0.17	0.17	−1.05	0.0001	Direct effect of wnt_score on GCG, independent of LYZ
LYZ	PYY	−0.19	0.21	−1.27	0.0001	Direct effect of wnt_score on PYY, independent of LYZ

## Data Availability

The data supporting the findings of this study are available from the corresponding authors upon request. All datasets used in this study are publicly available in established repositories. Accession numbers are provided in the Section 4.

## Supplementary Materials

The following supporting information can be downloaded at: https://www.mdpi.com/article/10.3390/ijms262211127/s1.

## Abbreviations

ACME	average causal mediation effect
ADE	average direct effect
AOM	azoxymethane
CAC	colitis-associated colorectal cancer
COAD	colon adenocarcinoma
CPTAC	National Cancer Institute’s Proteomics Tumor Analysis Consortium
CRC	colorectal cancer
DSS	dextran sulfate sodium
EEC	enteroendocrine cell
GEO	Gene Expression Omnibus
GO	Gene Ontology
GSEA	gene set enrichment analysis
IEC	intestinal epithelial cell
NCBI	National Center for Biotechnology Information
PCA	principal component analysis
PPI	protein–protein interaction
TCGA	The Cancer Genome Atlas Program
TME	tumor microenvironment

## References

[1] Bray F., Laversanne M., Sung H., Ferlay J., Siegel R.L., Soerjomataram I., Jemal A. (2024). Global Cancer Statistics 2022: GLOBOCAN Estimates of Incidence and Mortality Worldwide for 36 Cancers in 185 Countries. CA Cancer J. Clin..

[2] Terzić J., Grivennikov S., Karin E., Karin M. (2010). Inflammation and Colon Cancer. Gastroenterology.

[3] Bray G.A., Heisel W.E., Afshin A., Jensen M.D., Dietz W.H., Long M., Kushner R.F., Daniels S.R., Wadden T.A., Tsai A.G. (2018). The Science of Obesity Management: An Endocrine Society Scientific Statement. Endocr. Rev..

[4] Kolb R., Sutterwala F.S., Zhang W. (2016). Obesity and Cancer: Inflammation Bridges the Two. Curr. Opin. Pharmacol..

[5] Park S.-Y., Kim J.-S., Seo Y.-R., Sung M.-K. (2012). Effects of Diet-Induced Obesity on Colitis-Associated Colon Tumor Formation in A/J Mice. Int. J. Obes..

[6] Moghaddam A.A., Woodward M., Huxley R. (2007). Obesity and Risk of Colorectal Cancer: A Meta-Analysis of 31 Studies with 70,000 Events. Cancer Epidemiol. Biomark. Prev..

[7] Rustgi A.K. (2007). The Genetics of Hereditary Colon Cancer. Genes. Dev..

[8] Worthington J.J., Reimann F., Gribble F.M. (2018). Enteroendocrine Cells-Sensory Sentinels of the Intestinal Environment and Orchestrators of Mucosal Immunity. Mucosal Immunol..

[9] Li H.J., Ray S.K., Singh N.K., Johnston B., Leiter A.B. (2011). Basic Helix-loop-helix Transcription Factors and Enteroendocrine Cell Differentiation. Diabetes Obes. Metab..

[10] Bastide P., Darido C., Pannequin J., Kist R., Robine S., Marty-Double C., Bibeau F., Scherer G., Joubert D., Hollande F. (2007). Sox9 Regulates Cell Proliferation and Is Required for Paneth Cell Differentiation in the Intestinal Epithelium. J. Cell Biol..

[11] Hendel S.K., Kellermann L., Hausmann A., Bindslev N., Jensen K.B., Nielsen O.H. (2022). Tuft Cells and Their Role in Intestinal Diseases. Front. Immunol..

[12] Birchenough G.M.H., Johansson M.E., Gustafsson J.K., Bergström J.H., Hansson G.C. (2015). New Developments in Goblet Cell Mucus Secretion and Function. Mucosal Immunol..

[13] Koehne de Gonzalez A., del Portillo A. (2023). P754 Beyond Paneth Cell Metaplasia: Small Intestinal Metaplasia of the Sigmoid Colon in Patients with Inflammatory Bowel Disease. J. Crohns Colitis.

[14] Bevins C.L., Salzman N.H. (2011). Paneth Cells, Antimicrobial Peptides and Maintenance of Intestinal Homeostasis. Nat. Rev. Microbiol..

[15] Gunawardene A.R., Corfe B.M., Staton C.A. (2011). Classification and Functions of Enteroendocrine Cells of the Lower Gastrointestinal Tract. Int. J. Exp. Pathol..

[16] Drucker D.J. (2024). The GLP-1 Journey: From Discovery Science to Therapeutic Impact. J. Clin. Investig..

[17] Yu Y., Yang W., Li Y., Cong Y. (2020). Enteroendocrine Cells: Sensing Gut Microbiota and Regulating Inflammatory Bowel Diseases. Inflamm. Bowel Dis..

[18] da Silva E.M., Yariwake V.Y., Alves R.W., de Araujo D.R., Andrade-Oliveira V. (2022). Crosstalk between Incretin Hormones, Th17 and Treg Cells in Inflammatory Diseases. Peptides.

[19] Okayasu I., Ohkusa T., Kajiura K., Kanno J., Sakamoto S. (1996). Promotion of Colorectal Neoplasia in Experimental Murine Ulcerative Colitis. Gut.

[20] Tominaga O., Nita M.E., Nagawa H., Fujii S., Tsuruo T., Muto T. (1997). Expressions of Cell Cycle Regulators in Human Colorectal Cancer Cell Lines. Jpn. J. Cancer Res..

[21] Luvhengo T., Mabasa S., Molepo E., Taunyane I., Palweni S.T. (2023). Paneth Cell, Gut Microbiota Dysbiosis and Diabetes Mellitus. Appl. Sci..

[22] Wang L., Shen F., Stroehlein J.R., Wei D. (2018). Context-Dependent Functions of KLF4 in Cancers: Could Alternative Splicing Isoforms Be the Key?. Cancer Lett..

[23] Abdullayeva G., Liu H., Liu T.-C., Simmons A., Novelli M., Huseynova I., Lastun V.L., Bodmer W. (2024). Goblet Cell Differentiation Subgroups in Colorectal Cancer. Proc. Natl. Acad. Sci. USA.

[24] Habib A.M., Richards P., Cairns L.S., Rogers G.J., Bannon C.A.M., Parker H.E., Morley T.C.E., Yeo G.S.H., Reimann F., Gribble F.M. (2012). Overlap of Endocrine Hormone Expression in the Mouse Intestine Revealed by Transcriptional Profiling and Flow Cytometry. Endocrinology.

[25] Gierl M.S., Karoulias N., Wende H., Strehle M., Birchmeier C. (2006). The Zinc-Finger Factor Insm1 (IA-1) Is Essential for the Development of Pancreatic β Cells and Intestinal Endocrine Cells. Genes. Dev..

[26] Naya F.J., Huang H.-P., Qiu Y., Mutoh H., DeMayo F.J., Leiter A.B., Tsai M.-J. (1997). Diabetes, Defective Pancreatic Morphogenesis, and Abnormal Enteroendocrine Differentiation in BETA2/NeuroD-Deficient Mice. Genes. Dev..

[27] Deacon C.F. (2011). Dipeptidyl Peptidase-4 Inhibitors in the Treatment of Type 2 Diabetes: A Comparative Review. Diabetes Obes. Metab..

[28] Collombat P., Mansouri A., Hecksher-Sørensen J., Serup P., Krull J., Gradwohl G., Gruss P. (2003). Opposing Actions of Arx and Pax4 in Endocrine Pancreas Development. Genes. Dev..

[29] Estall J.L., Drucker D.J. (2006). Glucagon-Like Peptide-2. Annu. Rev. Nutr..

[30] Gradwohl G., Dierich A., LeMeur M., Guillemot F. (2000). *Neurogenin3* is Required for the Development of the Four Endocrine Cell Lineages of the Pancreas. Proc. Natl. Acad. Sci. USA.

[31] Gross S., Garofalo D.C., Balderes D.A., Mastracci T.L., Dias J.M., Perlmann T., Ericson J., Sussel L. (2016). Lmx1a Functions in Intestinal Serotonin-Producing Enterochromaffin Cells Downstream of Nkx2.2. Development.

[32] Katz J.P., Perreault N., Goldstein B.G., Lee C.S., Labosky P.A., Yang V.W., Kaestner K.H. (2002). The Zinc-Finger Transcription Factor Klf4 Is Required for Terminal Differentiation of Goblet Cells in the Colon. Development.

[33] Van der Sluis M., De Koning B.A.E., De Bruijn A.C.J.M., Velcich A., Meijerink J.P.P., Van Goudoever J.B., Büller H.A., Dekker J., Van Seuningen I., Renes I.B. (2006). Muc2-Deficient Mice Spontaneously Develop Colitis, Indicating That MUC2 Is Critical for Colonic Protection. Gastroenterology.

[34] Noah T.K., Kazanjian A., Whitsett J., Shroyer N.F. (2010). SAM Pointed Domain ETS Factor (SPDEF) Regulates Terminal Differentiation and Maturation of Intestinal Goblet Cells. Exp. Cell Res..

[35] Gerbe F., Sidot E., Smyth D.J., Ohmoto M., Matsumoto I., Dardalhon V., Cesses P., Garnier L., Pouzolles M., Brulin B. (2016). Intestinal Epithelial Tuft Cells Initiate Type 2 Mucosal Immunity to Helminth Parasites. Nature.

[36] Gracz A.D., Samsa L.A., Fordham M.J., Trotier D.C., Zwarycz B., Lo Y.-H., Bao K., Starmer J., Raab J.R., Shroyer N.F. (2018). Sox4 Promotes Atoh1-Independent Intestinal Secretory Differentiation Toward Tuft and Enteroendocrine Fates. Gastroenterology.

[37] Shroyer N.F., Wallis D., Venken K.J.T., Bellen H.J., Zoghbi H.Y. (2005). *Gfi1* Functions Downstream of *Math1* to Control Intestinal Secretory Cell Subtype Allocation and Differentiation. Genes. Dev..

[38] Valdez-Miramontes C.E., De Haro-Acosta J., Aréchiga-Flores C.F., Verdiguel-Fernández L., Rivas-Santiago B. (2021). Antimicrobial Peptides in Domestic Animals and Their Applications in Veterinary Medicine. Peptides.

[39] Yu S., Balasubramanian I., Laubitz D., Tong K., Bandyopadhyay S., Lin X., Flores J., Singh R., Liu Y., Macazana C. (2020). Paneth Cell-Derived Lysozyme Defines the Composition of Mucolytic Microbiota and the Inflammatory Tone of the Intestine. Immunity.

[40] Yu M., Qin K., Fan J., Zhao G., Zhao P., Zeng W., Chen C., Wang A., Wang Y., Zhong J. (2024). The Evolving Roles of Wnt Signaling in Stem Cell Proliferation and Differentiation, the Development of Human Diseases, and Therapeutic Opportunities. Genes. Dis..

[41] Barker N., van Es J.H., Kuipers J., Kujala P., van den Born M., Cozijnsen M., Haegebarth A., Korving J., Begthel H., Peters P.J. (2007). Identification of Stem Cells in Small Intestine and Colon by Marker Gene Lgr5. Nature.

[42] Ren J., Sui H., Fang F., Li Q., Li B. (2019). The Application of ApcMin/+ Mouse Model in Colorectal Tumor Researches. J. Cancer Res. Clin. Oncol..

[43] Amin M.B., Greene F.L., Edge S.B., Compton C.C., Gershenwald J.E., Brookland R.K., Meyer L., Gress D.M., Byrd D.R., Winchester D.P. (2017). The Eighth Edition AJCC Cancer Staging Manual: Continuing to Build a Bridge from a Population-based to a More “Personalized” Approach to Cancer Staging. CA Cancer J. Clin..

[44] Andreu P., Peignon G., Slomianny C., Taketo M.M., Colnot S., Robine S., Lamarque D., Laurent-Puig P., Perret C., Romagnolo B. (2008). A Genetic Study of the Role of the Wnt/β-Catenin Signalling in Paneth Cell Differentiation. Dev. Biol..

[45] Betge J., Schneider N.I., Harbaum L., Pollheimer M.J., Lindtner R.A., Kornprat P., Ebert M.P., Langner C. (2016). MUC1, MUC2, MUC5AC, and MUC6 in Colorectal Cancer: Expression Profiles and Clinical Significance. Virchows Arch..

[46] Westphalen C.B., Takemoto Y., Tanaka T., Macchini M., Jiang Z., Renz B.W., Chen X., Ormanns S., Nagar K., Tailor Y. (2016). Dclk1 Defines Quiescent Pancreatic Progenitors That Promote Injury-Induced Regeneration and Tumorigenesis. Cell Stem Cell.

[47] Yamada Y., Simon-Keller K., Belharazem-Vitacolonnna D., Bohnenberger H., Kriegsmann M., Kriegsmann K., Hamilton G., Graeter T., Preissler G., Ott G. (2021). A Tuft Cell–Like Signature Is Highly Prevalent in Thymic Squamous Cell Carcinoma and Delineates New Molecular Subsets Among the Major Lung Cancer Histotypes. J. Thorac. Oncol..

[48] Li L., Ma M., Duan T., Sui X. (2022). The Critical Roles and Therapeutic Implications of Tuft Cells in Cancer. Front. Pharmacol..

[49] Koehler J.A., Kain T., Drucker D.J. (2011). Glucagon-Like Peptide-1 Receptor Activation Inhibits Growth and Augments Apoptosis in Murine CT26 Colon Cancer Cells. Endocrinology.

[50] Koehler J.A., Baggio L.L., Yusta B., Longuet C., Rowland K.J., Cao X., Holland D., Brubaker P.L., Drucker D.J. (2015). GLP-1R Agonists Promote Normal and Neoplastic Intestinal Growth through Mechanisms Requiring Fgf7. Cell Metab..

[51] Zhang X., Zhang H., Shen B., Sun X.-F. (2019). Chromogranin-A Expression as a Novel Biomarker for Early Diagnosis of Colon Cancer Patients. Int. J. Mol. Sci..

[52] Zhang X., Zhang H., Fan C., Hildesjö C., Shen B., Sun X.-F. (2022). Loss of CHGA Protein as a Potential Biomarker for Colon Cancer Diagnosis: A Study on Biomarker Discovery by Machine Learning and Confirmation by Immunohistochemistry in Colorectal Cancer Tissue Microarrays. Cancers.

[53] Safarpour H., Ranjbaran J., Erfanian N., Nomiri S., Derakhshani A., Gerarduzzi C., Miraki Feriz A., HosseiniGol E., Saghafi S., Silvestris N. (2024). Holistic Exploration of CHGA and Hsa-MiR-137 in Colorectal Cancer via Multi-Omic Data Integration. Heliyon.

[54] Jing F., Liu G., Zhang R., Xue W., Lin J., Zhu H., Zhu Y., Wu C., Luo Y., Chen T. (2022). PYY Modulates the Tumorigenesis and Progression of Colorectal Cancer Unveiled by Proteomics. Am. J. Cancer Res..

[55] Lei K., Li W., Huang C., Li Y., Alfason L., Zhao H., Miyagishi M., Wu S., Kasim V. (2020). Neurogenic Differentiation Factor 1 Promotes Colorectal Cancer Cell Proliferation and Tumorigenesis by Suppressing the P53/P21 Axis. Cancer Sci..

[56] Li Z., He Y., Li Y., Li J., Zhao H., Song G., Miyagishi M., Wu S., Kasim V. (2021). NeuroD1 Promotes Tumor Cell Proliferation and Tumorigenesis by Directly Activating the Pentose Phosphate Pathway in Colorectal Carcinoma. Oncogene.

[57] Dai H., Li Y., Lee Y.A., Lu Y., George T.J., Donahoo W.T., Lee K.P., Nakshatri H., Allen J., Guo Y. (2025). GLP-1 Receptor Agonists and Cancer Risk in Adults With Obesity. JAMA Oncol..

[58] Shin J.H., Park J., Lim J., Jeong J., Dinesh R.K., Maher S.E., Kim J., Park S., Hong J.Y., Wysolmerski J. (2024). Metastasis of Colon Cancer Requires Dickkopf-2 to Generate Cancer Cells with Paneth Cell Properties. Elife.

[59] Pinto D., Gregorieff A., Begthel H., Clevers H. (2003). Canonical Wnt Signals Are Essential for Homeostasis of the Intestinal Epithelium. Genes. Dev..

[60] López-Arribillaga E., Yan B., Lobo-Jarne T., Guillén Y., Menéndez S., Andreu M., Bigas A., Iglesias M., Espinosa L. (2021). Accumulation of Paneth Cells in Early Colorectal Adenomas Is Associated with Beta-Catenin Signaling and Poor Patient Prognosis. Cells.

[61] Shen J., Wang Q., Mao Y., Gao W., Duan S. (2023). Targeting the P53 Signaling Pathway in Cancers: Molecular Mechanisms and Clinical Studies. MedComm.

[62] Du W., Miao Y., Zhang G., Luo G., Yang P., Chen F., Zhang B., Yang C., Li G., Chang J. (2022). The Regulatory Role of Neuropeptide Gene Glucagon in Colorectal Cancer: A Comprehensive Bioinformatic Analysis. Dis. Markers.

[63] Zhou G., Soufan O., Ewald J., Hancock R.E.W., Basu N., Xia J. (2019). NetworkAnalyst 3.0: A Visual Analytics Platform for Comprehensive Gene Expression Profiling and Meta-Analysis. Nucleic Acids Res..

[64] Zhou Y., Zhou B., Pache L., Chang M., Khodabakhshi A.H., Tanaseichuk O., Benner C., Chanda S.K. (2019). Metascape Provides a Biologist-Oriented Resource for the Analysis of Systems-Level Datasets. Nat. Commun..

[65] Tang G., Cho M., Wang X. (2022). OncoDB: An Interactive Online Database for Analysis of Gene Expression and Viral Infection in Cancer. Nucleic Acids Res..

[66] Chandrashekar D.S., Karthikeyan S.K., Korla P.K., Patel H., Shovon A.R., Athar M., Netto G.J., Qin Z.S., Kumar S., Manne U. (2022). UALCAN: An Update to the Integrated Cancer Data Analysis Platform. Neoplasia.

[67] Szklarczyk D., Franceschini A., Wyder S., Forslund K., Heller D., Huerta-Cepas J., Simonovic M., Roth A., Santos A., Tsafou K.P. (2015). STRING V10: Protein–Protein Interaction Networks, Integrated over the Tree of Life. Nucleic Acids Res..

[68] Uhlén M., Fagerberg L., Hallström B.M., Lindskog C., Oksvold P., Mardinoglu A., Sivertsson Å., Kampf C., Sjöstedt E., Asplund A. (2015). Tissue-Based Map of the Human Proteome. Science (1979).

[69] Fekete J.T., Győrffy B. (2019). ROCplot.Org: Validating Predictive Biomarkers of Chemotherapy/Hormonal Therapy/Anti-HER2 Therapy Using Transcriptomic Data of 3,104 Breast Cancer Patients. Int. J. Cancer.

