# Supplementary material for: Downregulation of Enteroendocrine Genes Predicts Survival in Colon Cancer: A Bioinformatics-Based Analysis
Source: Int J Mol Sci. 2025 Nov 18;26(22):11127. doi: 10.3390/ijms262211127 (PMC12652218; doi:10.3390/ijms262211127)
Supplement: Supplementary file 1 [file ijms-26-11127-s001.zip › Supplementary/Table_S1.pdf]

Table S1 - A total of 295 biological processes were found to be upregulated in mice, encompassing a comprehensive range of functional pathways.

Term	Description	LogP	Log(q-value)	Term	Description	LogP	Log(q-value)
GO:0006954	inflammatory response	-11,56281789	-7,372766469	GO:0007204	positive regulation of cytosolic calcium ion concentration	-2,559960216	-0,540170514
GO:0031347	regulation of defense response	-9,001475517	-5,112454095	GO:0031341	regulation of cell killing	-2,53061664	-0,51375149
GO:0030335	positive regulation of cell migration	-8,736408609	-5,023478446	GO:2000242	negative regulation of reproductive process	-2,522768826	-0,508808667
GO:0030595	leukocyte chemotaxis	-8,242409148	-4,89745577	GO:0030193	regulation of blood coagulation	-2,487590554	-0,476516083
GO:0040017	positive regulation of locomotion	-8,282462448	-4,89745577	GO:0002763	positive regulation of myeloid leukocyte differentiation	-2,470404005	-0,473477186
GO:0051272	positive regulation of cellular component movement	-8,272581046	-4,89745577	GO:0006721	terpenoid metabolic process	-2,470404005	-0,473477186
GO:2000147	positive regulation of cell motility	-8,453319101	-4,89745577	GO:0010632	regulation of epithelial cell migration	-2,473070315	-0,473477186
GO:0060326	cell chemotaxis	-7,781095231	-4,4941338	GO:0070613	regulation of protein processing	-2,470404005	-0,473477186
GO:0030593	neutrophil chemotaxis	-7,612445271	-4,376636362	GO:2000116	regulation of cysteine-type endopeptidase activity	-2,473070315	-0,473477186
GO:0050900	leukocyte migration	-7,513918248	-4,32386683	GO:0043408	regulation of MAPK cascade	-2,452419478	-0,463765184
GO:0071621	granulocyte chemotaxis	-7,336577567	-4,187918835	GO:1900046	regulation of hemostasis	-2,453475812	-0,463765184
GO:1990266	neutrophil migration	-7,119883165	-4,009012993	GO:1905330	regulation of morphogenesis of an epithelium	-2,453475812	-0,463765184
GO:0097530	granulocyte migration	-6,761599272	-3,685491207	GO:0050670	regulation of lymphocyte proliferation	-2,442634715	-0,45670328
GO:0050727	regulation of inflammatory response	-6,600408228	-3,556484845	GO:0002886	regulation of myeloid leukocyte mediated immunity	-2,420366239	-0,445158669
GO:0030162	regulation of proteolysis	-6,359767796	-3,389855393	GO:0030104	water homeostasis	-2,420366239	-0,445158669
GO:0032103	positive regulation of response to external stimulus	-6,349457889	-3,389855393	GO:0050818	regulation of coagulation	-2,420366239	-0,445158669
GO:0032963	collagen metabolic process	-6,370750063	-3,389855393	GO:1903317	regulation of protein maturation	-2,420366239	-0,445158669
GO:0010942	positive regulation of cell death	-6,013892128	-3,082344463	GO:0032944	regulation of mononuclear cell proliferation	-2,41278598	-0,440218507
GO:0097529	myeloid leukocyte migration	-5,99364228	-3,082344463	GO:0002694	regulation of leukocyte activation	-2,383771981	-0,419029845
GO:0006935	chemotaxis	-5,869436578	-3,004141709	GO:0010634	positive regulation of epithelial cell migration	-2,384673455	-0,419029845
GO:0030574	collagen catabolic process	-5,873671678	-3,004141709	GO:0022407	regulation of cell-cell adhesion	-2,36072261	-0,401120114
GO:0042330	taxis	-5,851770446	-3,004141709	GO:0045669	positive regulation of osteoblast differentiation	-2,356952027	-0,399896719
GO:0043068	positive regulation of programmed cell death	-5,778818471	-2,950494889	GO:0002274	myeloid leukocyte activation	-2,34571255	-0,393707235
GO:0045862	positive regulation of proteolysis	-5,632589649	-2,822749473	GO:0045667	regulation of osteoblast differentiation	-2,326649722	-0,377147552
GO:0035425	autocrine signaling	-5,414301912	-2,629252078	GO:0051249	regulation of lymphocyte activation	-2,292413903	-0,347875153
GO:0070098	chemokine-mediated signaling pathway	-5,404330148	-2,629252078	GO:0045824	negative regulation of innate immune response	-2,282465967	-0,340387815
GO:0002138	retinoic acid biosynthetic process	-5,0859302	-2,35827678	GO:0006959	humoral immune response	-2,258981354	-0,323526956
GO:0016102	diterpenoid biosynthetic process	-5,0859302	-2,35827678	GO:0072507	divalent inorganic cation homeostasis	-2,258305869	-0,323526956
GO:0043065	positive regulation of apoptotic process	-5,088816102	-2,35827678	GO:0031638	zymogen activation	-2,25402801	-0,323045415
GO:1990868	response to chemokine	-5,055557853	-2,356868129	GO:0071248	cellular response to metal ion	-2,253025445	-0,323045415
GO:1990869	cellular response to chemokine	-5,055557853	-2,356868129	GO:0042176	regulation of protein catabolic process	-2,206143763	-0,281691629
GO:0043568	positive regulation of insulin-like growth factor receptor signaling pathway	-4,948937931	-2,264036492	GO:0055082	cellular chemical homeostasis	-2,204571318	-0,281691629
GO:0002683	negative regulation of immune system process	-4,751822915	-2,080285437	GO:0002696	positive regulation of leukocyte activation	-2,187082295	-0,270121807
GO:0045670	regulation of osteoclast differentiation	-4,625762294	-1,967189793	GO:0050672	negative regulation of lymphocyte proliferation	-2,186015376	-0,270121807
GO:0002761	regulation of myeloid leukocyte differentiation	-4,597123868	-1,951140494	GO:0097190	apoptotic signaling pathway	-2,186656488	-0,270121807
GO:0034103	regulation of tissue remodeling	-4,550047911	-1,928198218	GO:0006720	isoprenoid metabolic process	-2,172909776	-0,263891726
GO:0150076	neuroinflammatory response	-4,555027179	-1,928198218	GO:0032945	negative regulation of mononuclear cell proliferation	-2,172909776	-0,263891726
GO:0016114	terpenoid biosynthetic process	-4,51365004	-1,914663229	GO:0051604	protein maturation	-2,173995974	-0,263891726
GO:0043584	nose development	-4,51365004	-1,914663229	GO:0030003	cellular cation homeostasis	-2,165084419	-0,262834731
GO:0120254	olefinic compound metabolic process	-4,443672407	-1,85568098	GO:0071222	cellular response to lipopolysaccharide	-2,167706613	-0,262834731
GO:0031349	positive regulation of defense response	-4,382304139	-1,805036578	GO:0043523	regulation of neuron apoptotic process	-2,15520872	-0,257413374
GO:0032102	negative regulation of response to external stimulus	-4,337019862	-1,770217735	GO:0032651	regulation of interleukin-1 beta production	-2,122025355	-0,228639127
GO:0002685	regulation of leukocyte migration	-4,316855143	-1,763371161	GO:0042445	hormone metabolic process	-2,117161451	-0,22596311
GO:0002687	positive regulation of leukocyte migration	-4,300210064	-1,763371161	GO:0010951	negative regulation of endopeptidase activity	-2,109127607	-0,224427559
GO:0019221	cytokine-mediated signaling pathway	-4,303292291	-1,763371161	GO:0014910	regulation of smooth muscle cell migration	-2,109672522	-0,224427559
GO:0045124	regulation of bone resorption	-4,185216594	-1,657923008	GO:0042129	regulation of T cell proliferation	-2,109127607	-0,224427559
GO:0002009	morphogenesis of an epithelium	-4,131439177	-1,620102097	GO:0071219	cellular response to molecule of bacterial origin	-2,106267527	-0,223712147
GO:0048729	tissue morphogenesis	-4,128912277	-1,620102097	GO:0001775	cell activation	-2,083458295	-0,221786481
GO:0001503	ossification	-4,099026564	-1,599171226	GO:0001818	negative regulation of cytokine production	-2,088335012	-0,221786481
GO:0048661	positive regulation of smooth muscle cell proliferation	-4,070518821	-1,579437408	GO:0002690	positive regulation of leukocyte chemotaxis	-2,08538748	-0,221786481
GO:0001817	regulation of cytokine production	-4,007129812	-1,533081738	GO:0002833	positive regulation of response to biotic stimulus	-2,101144095	-0,221786481
GO:0052547	regulation of peptidase activity	-4,014720526	-1,533081738	GO:0009615	response to virus	-2,094287617	-0,221786481
GO:0090026	positive regulation of monocyte chemotaxis	-3,993301599	-1,527526051	GO:0050867	positive regulation of cell activation	-2,098085847	-0,221786481
GO:0060541	respiratory system development	-3,960714187	-1,503056529	GO:0006873	cellular ion homeostasis	-2,081203085	-0,221565441
GO:0046850	regulation of bone remodeling	-3,884989112	-1,443125721	GO:0031214	biomineral tissue development	-2,061644362	-0,204031405
GO:0090303	positive regulation of wound healing	-3,884989112	-1,443125721	GO:0031343	positive regulation of cell killing	-2,049969254	-0,19837433
GO:0040013	negative regulation of locomotion	-3,865709852	-1,439086427	GO:0043502	regulation of muscle adaptation	-2,049969254	-0,19837433
GO:1903555	regulation of tumor necrosis factor superfamily cytokine production	-3,8682448	-1,439086427	GO:0071396	cellular response to lipid	-2,053276945	-0,19837433
GO:0043567	regulation of insulin-like growth factor receptor signaling pathway	-3,823017308	-1,406584768	GO:0001649	osteoblast differentiation	-2,038421662	-0,190792925
GO:0048771	tissue remodeling	-3,818484935	-1,406584768	GO:0097191	extrinsic apoptotic signaling pathway	-2,038421662	-0,190792925
GO:0002523	leukocyte migration involved in inflammatory response	-3,771078967	-1,400571485	GO:0002697	regulation of immune effector process	-2,015353813	-0,169694669
GO:0030198	extracellular matrix organization	-3,801008779	-1,400571485	GO:0061138	morphogenesis of a branching epithelium	-2,001704061	-0,158005618
GO:0043062	extracellular structure organization	-3,79138431	-1,400571485	GO:0048732	gland development	-1,99704634	-0,155588751
GO:0045229	external encapsulating structure organization	-3,781800015	-1,400571485	GO:0110148	biomineralization	-1,982511641	-0,14848608
GO:0045780	positive regulation of bone resorption	-3,771078967	-1,400571485	GO:0010595	positive regulation of endothelial cell migration	-1,971679217	-0,141463281
GO:0048286	lung alveolus development	-3,779235326	-1,400571485	GO:0032755	positive regulation of interleukin-6 production	-1,971679217	-0,141463281
GO:0043903	regulation of biological process involved in symbiotic interaction	-3,753875538	-1,389898923	GO:0032760	positive regulation of tumor necrosis factor production	-1,96095828	-0,136394847
GO:0042573	retinoic acid metabolic process	-3,721233396	-1,368932605	GO:0046889	positive regulation of lipid biosynthetic process	-1,96095828	-0,136394847
GO:0061844	antimicrobial humoral immune response mediated by antimicrobial peptide	-3,720134932	-1,368932605	GO:0022408	negative regulation of cell-cell adhesion	-1,95835	-0,135654504
GO:0071677	positive regulation of mononuclear cell migration	-3,704359383	-1,359406005	GO:0032642	regulation of chemokine production	-1,950346789	-0,129511228
GO:0001774	microglial cell activation	-3,627203969	-1,292024367	GO:0045639	positive regulation of myeloid cell differentiation	-1,939842758	-0,120859202
GO:0010038	response to metal ion	-3,624743288	-1,292024367	GO:2000241	regulation of reproductive process	-1,937231752	-0,120092337
GO:0090025	regulation of monocyte chemotaxis	-3,582752771	-1,256024213	GO:0001763	morphogenesis of a branching structure	-1,930272456	-0,115991488
GO:0061041	regulation of wound healing	-3,567121929	-1,246302231	GO:0030307	positive regulation of cell growth	-1,916471751	-0,104818234
GO:0008299	isoprenoid biosynthetic process	-3,539855182	-1,224865027	GO:1901615	organic hydroxy compound metabolic process	-1,912990414	-0,103150238
GO:0007162	negative regulation of cell adhesion	-3,486302543	-1,18274185	GO:0006875	cellular metal ion homeostasis	-1,904815617	-0,100370473
GO:0045637	regulation of myeloid cell differentiation	-3,488962887	-1,18274185	GO:0048562	embryonic organ morphogenesis	-1,904815617	-0,100370473
GO:0002269	leukocyte activation involved in inflammatory response	-3,458322001	-1,170179193	GO:0001558	regulation of cell growth	-1,891635092	-0,092518782
GO:0030336	negative regulation of cell migration	-3,461277987	-1,170179193	GO:0051251	positive regulation of lymphocyte activation	-1,891635092	-0,092518782
GO:1903036	positive regulation of response to wounding	-3,457140624	-1,170179193	GO:0032652	regulation of interleukin-1 production	-1,888868634	-0,09151417
GO:0050801	ion homeostasis	-3,430906003	-1,150742142	GO:0002832	negative regulation of response to biotic stimulus	-1,86916737	-0,077055961
GO:0070374	positive regulation of ERK1 and ERK2 cascade	-3,426979707	-1,150742142	GO:0051897	positive regulation of protein kinase B signaling	-1,86916737	-0,077055961
GO:0050729	positive regulation of inflammatory response	-3,412536992	-1,141563667	GO:0001910	regulation of leukocyte mediated cytotoxicity	-1,840311761	-0,053380864
GO:0030324	lung development	-3,396691832	-1,130919701	GO:0050866	negative regulation of cell activation	-1,843257379	-0,053380864
GO:0045672	positive regulation of osteoclast differentiation	-3,381897314	-1,121351456	GO:0072503	cellular divalent inorganic cation homeostasis	-1,830412535	-0,046901297
GO:0070372	regulation of ERK1 and ERK2 cascade	-3,371883621	-1,121351456	GO:0019725	cellular homeostasis	-1,811125743	-0,029314291
GO:0072330	monocarboxylic acid biosynthetic process	-3,372598808	-1,121351456	GO:0002688	regulation of leukocyte chemotaxis	-1,803073932	-0,022955637
GO:0030323	respiratory tube development	-3,366856667	-1,121287922	GO:0050920	regulation of chemotaxis	-1,798895488	-0,020463776
GO:2000146	negative regulation of cell motility	-3,356015718	-1,115354307	GO:0003073	regulation of systemic arterial blood pressure	-1,793975783	-0,018897713
GO:0061900	glial cell activation	-3,345413723	-1,109604815	GO:0043280	positive regulation of cysteine-type endopeptidase activity involved in apoptotic process	-1,793975783	-0,018897713
GO:0045088	regulation of innate immune response	-3,32776046	-1,101496869	GO:0002793	positive regulation of peptide secretion	-1,776023709	-0,004273582
GO:0046394	carboxylic acid biosynthetic process	-3,32776046	-1,101496869	GO:0001501	skeletal system development	-1,628986241	0
GO:0016053	organic acid biosynthetic process	-3,318105769	-1,101182204	GO:0001934	positive regulation of protein phosphorylation	-1,401937934	0
GO:0043525	positive regulation of neuron apoptotic process	-3,318331771	-1,101182204	GO:0002699	positive regulation of immune effector process	-1,399694108	0
GO:1904707	positive regulation of vascular associated smooth muscle cell proliferation	-3,309995664	-1,097667852	GO:0002700	regulation of production of molecular mediator of immune response	-1,422416695	0
GO:0051271	negative regulation of cellular component movement	-3,286013191	-1,078233006	GO:0003013	circulatory system process	-1,328638664	0
GO:0031348	negative regulation of defense response	-3,279952389	-1,076672706	GO:0006909	phagocytosis	-1,448479334	0
GO:1902105	regulation of leukocyte differentiation	-3,240577328	-1,041751986	GO:0007610	behavior	-1,495517349	0
GO:0019730	antimicrobial humoral response	-3,233430949	-1,039014726	GO:0007611	learning or memory	-1,340790037	0
GO:0042063	gliogenesis	-3,187694112	-0,997642694	GO:0007613	memory	-1,526223567	0
GO:0010817	regulation of hormone levels	-3,145144258	-0,963693012	GO:0008610	lipid biosynthetic process	-1,679162072	0
GO:0031076	embryonic camera-type eye development	-3,146995208	-0,963693012	GO:0009725	response to hormone	-1,345949401	0
GO:0070664	negative regulation of leukocyte proliferation	-3,139862154	-0,962647961	GO:0009896	positive regulation of catabolic process	-1,657430026	0
GO:0052548	regulation of endopeptidase activity	-3,123950385	-0,950932307	GO:0010001	glial cell differentiation	-1,302195442	0
GO:0002831	regulation of response to biotic stimulus	-3,074888252	-0,906026133	GO:0010466	negative regulation of peptidase activity	-1,620542291	0
GO:0032496	response to lipopolysaccharide	-3,054209729	-0,889464177	GO:0010594	regulation of endothelial cell migration	-1,441001653	0
GO:1903034	regulation of response to wounding	-3,048998055	-0,888330415	GO:0010950	positive regulation of endopeptidase activity	-1,466390031	0
GO:0070663	regulation of leukocyte proliferation	-3,040285693	-0,883658031	GO:0016331	morphogenesis of embryonic epithelium	-1,386363217	0
GO:0010952	positive regulation of peptidase activity	-3,027276273	-0,874651353	GO:0022409	positive regulation of cell-cell adhesion	-1,348997156	0
GO:0048660	regulation of smooth muscle cell proliferation	-3,016518452	-0,867859719	GO:0031331	positive regulation of cellular catabolic process	-1,382000514	0
GO:0032680	regulation of tumor necrosis factor production	-2,974156616	-0,829428177	GO:0031644	regulation of nervous system process	-1,31839018	0
GO:0001819	positive regulation of cytokine production	-2,899834573	-0,777969017	GO:0032675	regulation of interleukin-6 production	-1,479354327	0
GO:0002237	response to molecule of bacterial origin	-2,914989935	-0,777969017	GO:0032787	monocarboxylic acid metabolic process	-1,664635527	0
GO:0010035	response to inorganic substance	-2,899834573	-0,777969017	GO:0040008	regulation of growth	-1,468093403	0
GO:0050777	negative regulation of immune response	-2,902490602	-0,777969017	GO:0043583	ear development	-1,691985231	0
GO:0055080	cation homeostasis	-2,905271469	-0,777969017	GO:0044706	multi-multicellular organism process	-1,357397844	0
GO:1903557	positive regulation of tumor necrosis factor superfamily cytokine production	-2,906932831	-0,777969017	GO:0045089	positive regulation of innate immune response	-1,658725655	0
GO:1901214	regulation of neuron death	-2,876898368	-0,758728958	GO:0045834	positive regulation of lipid metabolic process	-1,357397844	0
GO:0008285	negative regulation of cell population proliferation	-2,853144885	-0,739754588	GO:0045861	negative regulation of proteolysis	-1,757034542	0
GO:0034754	cellular hormone metabolic process	-2,85062476	-0,739754588	GO:0045927	positive regulation of growth	-1,320563174	0
GO:0098771	inorganic ion homeostasis	-2,846206306	-0,738940259	GO:0048568	embryonic organ development	-1,65026264	0
GO:0044283	small molecule biosynthetic process	-2,821080447	-0,71738886	GO:0048592	eye morphogenesis	-1,39227184	0
GO:0030282	bone mineralization	-2,804982969	-0,71385797	GO:0048593	camera-type eye morphogenesis	-1,56836584	0
GO:0033993	response to lipid	-2,803538842	-0,71385797	GO:0048598	embryonic morphogenesis	-1,763541671	0
GO:0050886	endocrine process	-2,804982969	-0,71385797	GO:0048706	embryonic skeletal system development	-1,635324271	0
GO:0071675	regulation of mononuclear cell migration	-2,809792679	-0,71385797	GO:0048754	branching morphogenesis of an epithelial tube	-1,479354327	0
GO:0043410	positive regulation of MAPK cascade	-2,793888404	-0,707640707	GO:0050863	regulation of T cell activation	-1,724818834	0
GO:0042116	macrophage activation	-2,738425231	-0,662317166	GO:0050890	cognition	-1,76638821	0
GO:1902107	positive regulation of leukocyte differentiation	-2,740301995	-0,662317166	GO:0050921	positive regulation of chemotaxis	-1,597545662	0
GO:1903708	positive regulation of hemopoiesis	-2,740301995	-0,662317166	GO:0051047	positive regulation of secretion	-1,504534869	0
GO:0002820	negative regulation of adaptive immune response	-2,717061067	-0,650994398	GO:0051099	positive regulation of binding	-1,32385668	0
GO:0034341	response to interferon-gamma	-2,718823426	-0,650994398	GO:0051250	negative regulation of lymphocyte activation	-1,472849222	0
GO:0043281	regulation of cysteine-type endopeptidase activity involved in apoptotic process	-2,722231763	-0,650994398	GO:0051260	protein homooligomerization	-1,386363217	0
GO:0055065	metal ion homeostasis	-2,713941018	-0,650994398	GO:0051345	positive regulation of hydrolase activity	-1,601133808	0
GO:0007159	leukocyte cell-cell adhesion	-2,696084266	-0,639571757	GO:0051592	response to calcium ion	-1,767167237	0
GO:1903706	regulation of hemopoiesis	-2,696639363	-0,639571757	GO:0051896	regulation of protein kinase B signaling	-1,422416695	0
GO:0010812	negative regulation of cell-substrate adhesion	-2,675481792	-0,622150941	GO:0055074	calcium ion homeostasis	-1,768199084	0
GO:0016485	protein processing	-2,651898691	-0,603155793	GO:0060348	bone development	-1,756142426	0
GO:0071216	cellular response to biotic stimulus	-2,650192411	-0,603155793	GO:0060562	epithelial tube morphogenesis	-1,706765848	0
GO:1901216	positive regulation of neuron death	-2,64524553	-0,601322148	GO:0071241	cellular response to inorganic substance	-1,744209344	0
GO:0001523	retinoid metabolic process	-2,635350868	-0,594518563	GO:0071375	cellular response to peptide hormone stimulus	-1,302195442	0
GO:0014911	positive regulation of smooth muscle cell migration	-2,615799414	-0,578036341	GO:1901215	negative regulation of neuron death	-1,609943751	0
GO:0016101	diterpenoid metabolic process	-2,596576204	-0,567892789	GO:1903037	regulation of leukocyte cell-cell adhesion	-1,743129282	0
GO:0035821	modulation of process of another organism	-2,596576204	-0,567892789	GO:1903038	negative regulation of leukocyte cell-cell adhesion	-1,635324271	0
GO:0045732	positive regulation of protein catabolic process	-2,601094712	-0,567892789	GO:1903364	positive regulation of cellular protein catabolic process	-1,627648426	0
GO:0010810	regulation of cell-substrate adhesion	-2,584512896	-0,558814334	GO:2001056	positive regulation of cysteine-type endopeptidase activity	-1,612478554	0
GO:1904705	regulation of vascular associated smooth muscle cell proliferation	-2,577671048	-0,554936965	GO:2001233	regulation of apoptotic signaling pathway	-1,570975591	0
				GO:2001242	regulation of intrinsic apoptotic signaling pathway	-1,422416695	0
