# Supplementary material for: Downregulation of Enteroendocrine Genes Predicts Survival in Colon Cancer: A Bioinformatics-Based Analysis
Source: Int J Mol Sci. 2025 Nov 18;26(22):11127. doi: 10.3390/ijms262211127 (PMC12652218; doi:10.3390/ijms262211127)
Supplement: Supplementary file 1 [file ijms-26-11127-s001.zip › Supplementary/Table_S3.pdf]

Table S3 - In humans, 157 biological processes were identified as being upregulated, reflecting a diverse array of molecular and cellular pathways.

Term	Description	LogP	Log(q-value)	Term	Description	LogP	Log(q-value)
GO:1903047	mitotic cell cycle process	-12,24701142	-8,059	GO:0019730	antimicrobial humoral response	-2,900998677	-0,616
GO:0000278	mitotic cell cycle	-11,29746645	-7,587	GO:0071482	cellular response to light stimulus	-2,867987405	-0,589
GO:0010564	regulation of cell cycle process	-11,34314068	-7,587	GO:0043588	skin development	-2,835094384	-0,561
GO:0000280	nuclear division	-10,8679809	-7,282	GO:0007093	mitotic cell cycle checkpoint signaling	-2,784246307	-0,516
GO:0140014	mitotic nuclear division	-10,50796567	-7,097	GO:0097529	myeloid leukocyte migration	-2,764216436	-0,501
GO:0048285	organelle fission	-10,50686204	-7,097	GO:0006281	DNA repair	-2,656402174	-0,398
GO:0051301	cell division	-9,843050961	-6,5	GO:0044089	positive regulation of cellular component biogenesis	-2,629487821	-0,376
GO:0098813	nuclear chromosome segregation	-9,349668978	-6,065	GO:0006974	cellular response to DNA damage stimulus	-2,618639251	-0,37
GO:1905818	regulation of chromosome separation	-8,824966401	-5,592	GO:0030595	leukocyte chemotaxis	-2,580944273	-0,338
GO:0000070	mitotic sister chromatid segregation	-8,557114185	-5,369	GO:0009411	response to UV	-2,555811161	-0,318
GO:0007059	chromosome segregation	-8,499031549	-5,353	GO:0140694	non-membrane-bounded organelle assembly	-2,541982875	-0,309
GO:1901989	positive regulation of cell cycle phase transition	-8,431937048	-5,323	GO:0071214	cellular response to abiotic stimulus	-2,527090006	-0,303
GO:0051983	regulation of chromosome segregation	-8,288551723	-5,215	GO:0104004	cellular response to environmental stimulus	-2,527090006	-0,303
GO:0051303	establishment of chromosome localization	-8,166912988	-5,125	GO:0000075	cell cycle checkpoint signaling	-2,515117406	-0,302
GO:0050000	chromosome localization	-8,098176882	-5,087	GO:0006954	inflammatory response	-2,515449258	-0,302
GO:0000819	sister chromatid segregation	-7,937830331	-4,954	GO:0032496	response to lipopolysaccharide	-2,512356015	-0,302
GO:1901987	regulation of cell cycle phase transition	-7,910828019	-4,954	GO:0008285	negative regulation of cell population proliferation	-2,445647448	-0,24
GO:1901990	regulation of mitotic cell cycle phase transition	-7,673302485	-4,764	GO:0002237	response to molecule of bacterial origin	-2,41342468	-0,213
GO:0030071	regulation of mitotic metaphase/anaphase transition	-7,692196683	-4,764	GO:0030335	positive regulation of cell migration	-2,389329686	-0,193
GO:0033044	regulation of chromosome organization	-7,638863933	-4,752	GO:0051347	positive regulation of transferase activity	-2,340990827	-0,149
GO:1902099	regulation of metaphase/anaphase transition of cell cycle	-7,607185933	-4,742	GO:1901991	negative regulation of mitotic cell cycle phase transition	-2,331139885	-0,143
GO:0010965	regulation of mitotic sister chromatid separation	-7,55208194	-4,707	GO:0071478	cellular response to radiation	-2,324375655	-0,141
GO:0090068	positive regulation of cell cycle process	-7,519920216	-4,694	GO:2000147	positive regulation of cell motility	-2,312705428	-0,134
GO:1901992	positive regulation of mitotic cell cycle phase transition	-7,419436985	-4,612	GO:0051272	positive regulation of cellular component movement	-2,272759394	-0,098
GO:0033045	regulation of sister chromatid segregation	-7,3181978	-4,528	GO:0040017	positive regulation of locomotion	-2,26670679	-0,096
GO:0051310	metaphase plate congression	-6,995633325	-4,223	GO:0051302	regulation of cell division	-2,25891978	-0,092
GO:0045931	positive regulation of mitotic cell cycle	-6,784972722	-4,029	GO:2000241	regulation of reproductive process	-2,246283595	-0,084
GO:0045787	positive regulation of cell cycle	-6,444328178	-3,704	GO:0050678	regulation of epithelial cell proliferation	-2,231009438	-0,073
GO:0007346	regulation of mitotic cell cycle	-6,291339892	-3,566	GO:0006260	DNA replication	-2,197151546	-0,043
GO:0030574	collagen catabolic process	-6,095601545	-3,385	GO:0071222	cellular response to lipopolysaccharide	-2,191163051	-0,041
GO:0007052	mitotic spindle organization	-6,014632359	-3,318	GO:0006302	double-strand break repair	-2,185207417	-0,039
GO:0071492	cellular response to UV-A	-5,982644067	-3,3	GO:0060326	cell chemotaxis	-2,150145655	-0,008
GO:0022617	extracellular matrix disassembly	-5,755942473	-3,087	GO:0060249	anatomical structure homeostasis	-1,800897484	0
GO:0070141	response to UV-A	-5,640774339	-2,985	GO:1901137	carbohydrate derivative biosynthetic process	-1,609936311	0
GO:0007080	mitotic metaphase plate congression	-5,608766572	-2,965	GO:0046394	carboxylic acid biosynthetic process	-1,855856027	0
GO:1902850	microtubule cytoskeleton organization involved in mitosis	-5,403241944	-2,772	GO:0022412	cellular process involved in reproduction in multicellular organism	-1,393612533	0
GO:0032963	collagen metabolic process	-5,348292846	-2,729	GO:0071216	cellular response to biotic stimulus	-1,980391527	0
GO:0007096	regulation of exit from mitosis	-5,292515327	-2,685	GO:0071345	cellular response to cytokine stimulus	-1,964816968	0
GO:0007051	spindle organization	-4,947937387	-2,351	GO:0071363	cellular response to growth factor stimulus	-1,928700071	0
GO:0051984	positive regulation of chromosome segregation	-4,846553308	-2,261	GO:0071241	cellular response to inorganic substance	-2,07788485	0
GO:0022411	cellular component disassembly	-4,696706799	-2,122	GO:0071219	cellular response to molecule of bacterial origin	-2,121773782	0
GO:0071396	cellular response to lipid	-4,357312176	-1,803	GO:1901699	cellular response to nitrogen compound	-1,486226468	0
GO:0032506	cytokinetic process	-4,360983848	-1,803	GO:0019221	cytokine-mediated signaling pathway	-1,513930418	0
GO:0000226	microtubule cytoskeleton organization	-4,23510526	-1,691	GO:0008544	epidermis development	-1,706724254	0
GO:0051656	establishment of organelle localization	-4,131617487	-1,603	GO:0048732	gland development	-1,393612533	0
GO:0007088	regulation of mitotic nuclear division	-4,127441871	-1,603	GO:0006959	humoral immune response	-1,752697565	0
GO:0006259	DNA metabolic process	-4,052331935	-1,537	GO:0050900	leukocyte migration	-2,025452165	0
GO:0030198	extracellular matrix organization	-3,794708362	-1,302	GO:0007018	microtubule-based movement	-1,469119466	0
GO:0043062	extracellular structure organization	-3,787271994	-1,302	GO:0002009	morphogenesis of an epithelium	-1,996114098	0
GO:0033047	regulation of mitotic sister chromatid segregation	-3,806846422	-1,302	GO:0048871	multicellular organismal homeostasis	-1,504792959	0
GO:0051783	regulation of nuclear division	-3,781720259	-1,302	GO:0045786	negative regulation of cell cycle	-1,469119466	0
GO:0045229	external encapsulating structure organization	-3,772488376	-1,301	GO:1901988	negative regulation of cell cycle phase transition	-1,98530377	0
GO:0070542	response to fatty acid	-3,631463292	-1,168	GO:0010948	negative regulation of cell cycle process	-1,768507579	0
GO:0140013	meiotic nuclear division	-3,557204042	-1,102	GO:0051129	negative regulation of cellular component organization	-1,379195996	0
GO:1902808	positive regulation of cell cycle G1/S phase transition	-3,533300618	-1,086	GO:0045930	negative regulation of mitotic cell cycle	-2,040911296	0
GO:0061844	antimicrobial humoral immune response mediated by antimicrobial peptide	-3,477964074	-1,038	GO:0010639	negative regulation of organelle organization	-1,570712785	0
GO:0051640	organelle localization	-3,449757122	-1,018	GO:0016053	organic acid biosynthetic process	-1,847204671	0
GO:1903046	meiotic cell cycle process	-3,405661808	-0,981	GO:0033674	positive regulation of kinase activity	-1,864798343	0
GO:0001942	hair follicle development	-3,374285509	-0,957	GO:0045860	positive regulation of protein kinase activity	-2,128162482	0
GO:0000281	mitotic cytokinesis	-3,357832291	-0,948	GO:0001934	positive regulation of protein phosphorylation	-1,890830484	0
GO:0030593	neutrophil chemotaxis	-3,341598875	-0,939	GO:0071902	positive regulation of protein serine/threonine kinase activity	-2,05659966	0
GO:0022405	hair cycle process	-3,309769249	-0,928	GO:0030155	regulation of cell adhesion	-1,790125435	0
GO:0022404	molting cycle process	-3,309769249	-0,928	GO:1902806	regulation of cell cycle G1/S phase transition	-2,116188212	0
GO:0098773	skin epidermis development	-3,309769249	-0,928	GO:0042176	regulation of protein catabolic process	-1,407127146	0
GO:0070098	chemokine-mediated signaling pathway	-3,294162456	-0,919	GO:0045859	regulation of protein kinase activity	-1,444541572	0
GO:0071621	granulocyte chemotaxis	-3,263539904	-0,895	GO:0071900	regulation of protein serine/threonine kinase activity	-1,431977642	0
GO:1990869	cellular response to chemokine	-3,176074483	-0,842	GO:0043254	regulation of protein-containing complex assembly	-1,380299357	0
GO:0034644	cellular response to UV	-3,176074483	-0,842	GO:0009617	response to bacterium	-1,307004898	0
GO:0007017	microtubule-based process	-3,172403872	-0,842	GO:0070848	response to growth factor	-1,83841201	0
GO:1990266	neutrophil migration	-3,19021808	-0,842	GO:0009725	response to hormone	-1,863230465	0
GO:0050679	positive regulation of epithelial cell proliferation	-3,172116786	-0,842	GO:0009416	response to light stimulus	-1,688183762	0
GO:1990868	response to chemokine	-3,176074483	-0,842	GO:1901652	response to peptide	-2,016066817	0
GO:0042633	hair cycle	-3,121109294	-0,808	GO:0009314	response to radiation	-2,036342404	0
GO:0042303	molting cycle	-3,121109294	-0,808	GO:0009611	response to wounding	-1,309245183	0
GO:0032465	regulation of cytokinesis	-3,121109294	-0,808	GO:0044283	small molecule biosynthetic process	-1,321517127	0
GO:0097530	granulocyte migration	-3,081479632	-0,775	GO:0001894	tissue homeostasis	-2,00018022	0
GO:0061640	cytoskeleton-dependent cytokinesis	-2,969930449	-0,669	GO:0048729	tissue morphogenesis	-1,678647272	0
GO:0000910	cytokinesis	-2,946503514	-0,656	GO:0042060	wound healing	-1,610555049	0
GO:0051321	meiotic cell cycle	-2,950090572	-0,656				
