# Supplementary material for: Downregulation of Enteroendocrine Genes Predicts Survival in Colon Cancer: A Bioinformatics-Based Analysis
Source: Int J Mol Sci. 2025 Nov 18;26(22):11127. doi: 10.3390/ijms262211127 (PMC12652218; doi:10.3390/ijms262211127)
Supplement: Supplementary file 1 [file ijms-26-11127-s001.zip › Supplementary/Table_S2.pdf]

**Table S2 - A total of 66 biological processes were identified as downregulated in mice, representing a diverse set of functional pathways.**

| Term | Description | LogP | Log(q-value) |
|---|---|---|---|
| GO:1901615 | organic hydroxy compound metabolic process | -6,95471 | -2,765 |
| GO:0032787 | monocarboxylic acid metabolic process | -5,40678 | -1,518 |
| GO:0006066 | alcohol metabolic process | -4,71184 | -1,207 |
| GO:0006790 | sulfur compound metabolic process | -4,6184 | -1,207 |
| GO:0009410 | response to xenobiotic stimulus | -4,62604 | -1,207 |
| GO:0044282 | small molecule catabolic process | -4,63371 | -1,207 |
| GO:0006584 | catecholamine metabolic process | -4,08231 | -0,795 |
| GO:0009712 | catechol-containing compound metabolic process | -4,08231 | -0,795 |
| GO:0008202 | steroid metabolic process | -3,87815 | -0,642 |
| GO:1901617 | organic hydroxy compound biosynthetic process | -3,59885 | -0,409 |
| GO:0014070 | response to organic cyclic compound | -3,42307 | -0,361 |
| GO:0016054 | organic acid catabolic process | -3,29545 | -0,361 |
| GO:0034308 | primary alcohol metabolic process | -3,39998 | -0,361 |
| GO:0042445 | hormone metabolic process | -3,43079 | -0,361 |
| GO:0044242 | cellular lipid catabolic process | -3,40449 | -0,361 |
| GO:0046395 | carboxylic acid catabolic process | -3,31173 | -0,361 |
| GO:0051384 | response to glucocorticoid | -3,32075 | -0,361 |
| GO:0006631 | fatty acid metabolic process | -3,26034 | -0,349 |
| GO:0006576 | cellular biogenic amine metabolic process | -3,20386 | -0,342 |
| GO:0031960 | response to corticosteroid | -3,21787 | -0,342 |
| GO:0044106 | cellular amine metabolic process | -3,19001 | -0,342 |
| GO:0009308 | amine metabolic process | -3,12307 | -0,318 |
| GO:0018958 | phenol-containing compound metabolic process | -3,11012 | -0,318 |
| GO:0046165 | alcohol biosynthetic process | -3,12307 | -0,318 |
| GO:0044283 | small molecule biosynthetic process | -3,07799 | -0,303 |
| GO:0072329 | monocarboxylic acid catabolic process | -3,04738 | -0,289 |
| GO:0034754 | cellular hormone metabolic process | -2,89823 | -0,155 |
| GO:0009636 | response to toxic substance | -2,84552 | -0,118 |
| GO:0016042 | lipid catabolic process | -2,78047 | -0,068 |
| GO:0006091 | generation of precursor metabolites and energy | -1,63548 | 0 |
| GO:0006520 | cellular amino acid metabolic process | -1,96042 | 0 |
| GO:0006753 | nucleoside phosphate metabolic process | -1,34907 | 0 |
| GO:0006812 | cation transport | -1,93696 | 0 |
| GO:0006814 | sodium ion transport | -2,38412 | 0 |
| GO:0006816 | calcium ion transport | -2,07881 | 0 |
| GO:0006820 | anion transport | -1,42415 | 0 |
| GO:0006873 | cellular ion homeostasis | -1,37681 | 0 |
| GO:0008610 | lipid biosynthetic process | -1,83416 | 0 |
| GO:0009117 | nucleotide metabolic process | -1,36916 | 0 |
| GO:0009259 | ribonucleotide metabolic process | -1,58284 | 0 |
| GO:0009725 | response to hormone | -2,2711 | 0 |
| GO:0009991 | response to extracellular stimulus | -1,42146 | 0 |
| GO:0010038 | response to metal ion | -1,76931 | 0 |
| GO:0010817 | regulation of hormone levels | -2,38669 | 0 |
| GO:0019216 | regulation of lipid metabolic process | -1,46558 | 0 |
| GO:0019693 | ribose phosphate metabolic process | -1,54518 | 0 |
| GO:0019725 | cellular homeostasis | -1,56482 | 0 |
| GO:0030001 | metal ion transport | -2,34002 | 0 |
| GO:0030003 | cellular cation homeostasis | -1,42146 | 0 |
| GO:0031667 | response to nutrient levels | -1,5149 | 0 |
| GO:0032271 | regulation of protein polymerization | -2,1886 | 0 |
| GO:0033993 | response to lipid | -1,85336 | 0 |
| GO:0034220 | ion transmembrane transport | -2,23063 | 0 |
| GO:0043254 | regulation of protein-containing complex assembly | -1,36157 | 0 |
| GO:0043603 | cellular amide metabolic process | -1,8381 | 0 |
| GO:0048545 | response to steroid hormone | -2,47756 | 0 |
| GO:0048871 | multicellular organismal homeostasis | -1,34659 | 0 |
| GO:0050801 | ion homeostasis | -1,79283 | 0 |
| GO:0055080 | cation homeostasis | -1,86252 | 0 |
| GO:0055082 | cellular chemical homeostasis | -1,8147 | 0 |
| GO:0071466 | cellular response to xenobiotic stimulus | -2,37041 | 0 |
| GO:0072521 | purine-containing compound metabolic process | -1,5269 | 0 |
| GO:0097305 | response to alcohol | -2,05814 | 0 |
| GO:0097435 | supramolecular fiber organization | -1,75027 | 0 |
| GO:0098660 | inorganic ion transmembrane transport | -2,6397 | 0 |
| GO:0098771 | inorganic ion homeostasis | -1,83136 | 0 |
