# Supplementary material for: Downregulation of Enteroendocrine Genes Predicts Survival in Colon Cancer: A Bioinformatics-Based Analysis
Source: Int J Mol Sci. 2025 Nov 18;26(22):11127. doi: 10.3390/ijms262211127 (PMC12652218; doi:10.3390/ijms262211127)
Supplement: Supplementary file 1 [file ijms-26-11127-s001.zip › Supplementary/Table_S5.pdf]

Table S5 - Association between intestinal epithelial cell marker gene expression and colorectal cancer patient survival.

Cell type	Gene	Low expression n=	High expression n=	p score	5-year survival high (%)	5-year survival low (%)
Enteroendrine cells	PYY	157	97	0.34	75	57
	INSM1	75	179	0.21	67	56
	NEUROD1	132	122	0.23	55	70
	DPP4	86	168	0.19	71	52
	ARX	127	127	0.24	66	61
	NKX2.2	130	124	0.58	73	57
	GLP2R	126	128	0.10	66	60
	PAX6	130	124	0.55	64	63
	GIPR	109	145	0.13	58	71
	NEUROG3	129	125	0.35	71	58
	PAX4	122	132	0.78	64	62
	LMX1A	45	43	0.57	44	46
	GLP1R	109	45	0.23	57	70
	ISL1	126	128	0.51	61	65
	GIP	Not available data				
Goblet cells	KLF4	191	63	0.0051	91	57
	MUC2	112	142	0.012	77	44
	SPDEF	51	203	0.13	63	66
Tuft cells	POU2F3	127	127	0.70	57	68
	SOX4	118	136	0.13	61	62
Paneth cells	LYZ	125	129	0.46	60	67
	DEFA1	125	129	0.46	66	61
	GFI1	101	153	0.0013	75	50
	SOX9	109	145	0.19	64	62
	WISP1	103	151	0.14	50	74
Stem cell	LGR5	135	119	0.45	67	60
