# Supplementary material for: Downregulation of Enteroendocrine Genes Predicts Survival in Colon Cancer: A Bioinformatics-Based Analysis
Source: Int J Mol Sci. 2025 Nov 18;26(22):11127. doi: 10.3390/ijms262211127 (PMC12652218; doi:10.3390/ijms262211127)
Supplement: Supplementary file 1 [file ijms-26-11127-s001.zip › Supplementary/Table_S4.pdf]

Table S4 - A total of 167 biological processes were found to be downregulated in humans, encompassing a broad range of molecular and cellular pathways.

Termo	Descrição	LogP	Log(q-value)	Termo	Descrição	LogP	Log(q-value)
GO:0055080	cation homeostasis	-8,14409	-4,458	GO:0046903	secretion	-2,49623	-0,229
GO:0030003	cellular cation homeostasis	-8,27834	-4,458	GO:0032787	monocarboxylic acid metabolic process	-2,48652	-0,228
GO:0006873	cellular ion homeostasis	-8,08534	-4,458	GO:0090066	regulation of anatomical structure size	-2,47206	-0,219
GO:0098771	inorganic ion homeostasis	-8,04347	-4,458	GO:0070848	response to growth factor	-2,45772	-0,214
GO:0050801	ion homeostasis	-7,859	-4,37	GO:0071559	response to transforming growth factor beta	-2,45751	-0,214
GO:0055082	cellular chemical homeostasis	-7,75375	-4,344	GO:0031344	regulation of cell projection organization	-2,42234	-0,197
GO:0019725	cellular homeostasis	-6,81482	-3,472	GO:0031960	response to corticosteroid	-2,4284	-0,197
GO:0032870	cellular response to hormone stimulus	-6,36491	-3,13	GO:0003018	vascular process in circulatory system	-2,42061	-0,197
GO:0006730	one-carbon metabolic process	-6,36346	-3,13	GO:0098655	cation transmembrane transport	-2,41023	-0,191
GO:0009755	hormone-mediated signaling pathway	-6,15169	-2,964	GO:0042180	cellular ketone metabolic process	-2,37086	-0,156
GO:0006820	anion transport	-5,79692	-2,688	GO:0040008	regulation of growth	-2,33523	-0,13
GO:0009725	response to hormone	-5,81811	-2,688	GO:2000134	negative regulation of G1/S transition of mitotic cell cycle	-2,32772	-0,127
GO:0034308	primary alcohol metabolic process	-5,68826	-2,615	GO:0051591	response to cAMP	-2,2862	-0,094
GO:0006875	cellular metal ion homeostasis	-5,35428	-2,343	GO:0008015	blood circulation	-2,27509	-0,087
GO:0010817	regulation of hormone levels	-5,3662	-2,343	GO:0044106	cellular amine metabolic process	-2,25936	-0,083
GO:0008202	steroid metabolic process	-5,1309	-2,147	GO:0006576	cellular biogenic amine metabolic process	-2,25936	-0,083
GO:0031667	response to nutrient levels	-4,97807	-2,021	GO:0010959	regulation of metal ion transport	-2,24978	-0,079
GO:0042445	hormone metabolic process	-4,85998	-1,951	GO:0040013	negative regulation of locomotion	-2,23478	-0,07
GO:0048545	response to steroid hormone	-4,86929	-1,951	GO:0090087	regulation of peptide transport	-2,23231	-0,07
GO:1904995	negative regulation of leukocyte adhesion to vascular endothelial cell	-4,79141	-1,925	GO:0006874	cellular calcium ion homeostasis	-2,21585	-0,069
GO:1901615	organic hydroxy compound metabolic process	-4,79004	-1,925	GO:2000045	regulation of G1/S transition of mitotic cell cycle	-2,21585	-0,069
GO:0009991	response to extracellular stimulus	-4,71167	-1,866	GO:0043269	regulation of ion transport	-2,2153	-0,069
GO:0055067	monovalent inorganic cation homeostasis	-4,61395	-1,788	GO:0015718	monocarboxylic acid transport	-2,18261	-0,044
GO:0072503	cellular divalent inorganic cation homeostasis	-4,57976	-1,772	GO:1902807	negative regulation of cell cycle G1/S phase transition	-2,18261	-0,044
GO:0055065	metal ion homeostasis	-4,54931	-1,76	GO:0071345	cellular response to cytokine stimulus	-2,16519	-0,042
GO:0006066	alcohol metabolic process	-4,44951	-1,677	GO:1990830	cellular response to leukemia inhibitory factor	-2,15819	-0,042
GO:0006885	regulation of pH	-4,37148	-1,615	GO:0140352	export from cell	-2,1429	-0,042
GO:0006821	chloride transport	-4,26795	-1,548	GO:0007162	negative regulation of cell adhesion	-2,15244	-0,042
GO:0015698	inorganic anion transport	-4,25814	-1,548	GO:0051047	positive regulation of secretion	-2,16417	-0,042
GO:0097501	stress response to metal ion	-4,26889	-1,548	GO:0008217	regulation of blood pressure	-2,17559	-0,042
GO:0072507	divalent inorganic cation homeostasis	-4,20607	-1,51	GO:0010975	regulation of neuron projection development	-2,1429	-0,042
GO:0030004	cellular monovalent inorganic cation homeostasis	-4,11317	-1,435	GO:1990823	response to leukemia inhibitory factor	-2,14618	-0,042
GO:0043434	response to peptide hormone	-4,10405	-1,435	GO:0030855	epithelial cell differentiation	-2,12015	-0,026
GO:0032094	response to food	-4,01195	-1,356	GO:0098662	inorganic cation transmembrane transport	-2,11622	-0,025
GO:0034754	cellular hormone metabolic process	-3,85335	-1,21	GO:0042886	amide transport	-1,89261	0
GO:0098754	detoxification	-3,75881	-1,127	GO:0055074	calcium ion homeostasis	-2,04029	0
GO:0015701	bicarbonate transport	-3,70483	-1,097	GO:0019722	calcium-mediated signaling	-1,71272	0
GO:0010447	response to acidic pH	-3,70483	-1,097	GO:0032869	cellular response to insulin stimulus	-1,68187	0
GO:0019932	second-messenger-mediated signaling	-3,66388	-1,067	GO:0071396	cellular response to lipid	-1,8449	0
GO:0015711	organic anion transport	-3,60785	-1,022	GO:0071248	cellular response to metal ion	-1,4127	0
GO:0071383	cellular response to steroid hormone stimulus	-3,55551	-0,981	GO:0007167	enzyme-linked receptor protein signaling pathway	-1,49562	0
GO:0009636	response to toxic substance	-3,53847	-0,974	GO:0050900	leukocyte migration	-1,89265	0
GO:0051453	regulation of intracellular pH	-3,49372	-0,94	GO:0010876	lipid localization	-1,8967	0
GO:0071375	cellular response to peptide hormone stimulus	-3,44925	-0,924	GO:0006869	lipid transport	-2,07847	0
GO:0006882	cellular zinc ion homeostasis	-3,45911	-0,924	GO:0030001	metal ion transport	-1,97346	0
GO:1901652	response to peptide	-3,45549	-0,924	GO:0003012	muscle system process	-1,60285	0
GO:0030641	regulation of cellular pH	-3,39679	-0,881	GO:0097529	myeloid leukocyte migration	-1,88328	0
GO:1900542	regulation of purine nucleotide metabolic process	-3,37812	-0,872	GO:0010721	negative regulation of cell development	-1,46545	0
GO:0055069	zinc ion homeostasis	-3,35259	-0,855	GO:0030308	negative regulation of cell growth	-1,43577	0
GO:0006140	regulation of nucleotide metabolic process	-3,34146	-0,853	GO:0030336	negative regulation of cell migration	-1,84422	0
GO:0071417	cellular response to organonitrogen compound	-3,32968	-0,85	GO:2000146	negative regulation of cell motility	-1,77585	0
GO:1904994	regulation of leukocyte adhesion to vascular endothelial cell	-3,31906	-0,847	GO:0022408	negative regulation of cell-cell adhesion	-1,36289	0
GO:0009308	amine metabolic process	-3,25351	-0,79	GO:0051271	negative regulation of cellular component movement	-1,7408	0
GO:1900371	regulation of purine nucleotide biosynthetic process	-3,19355	-0,738	GO:1903038	negative regulation of leukocyte cell-cell adhesion	-1,72059	0
GO:0030808	regulation of nucleotide biosynthetic process	-3,16411	-0,717	GO:1901991	negative regulation of mitotic cell cycle phase transition	-1,48374	0
GO:0009268	response to pH	-3,13538	-0,696	GO:0051961	negative regulation of nervous system development	-1,71272	0
GO:0003013	circulatory system process	-3,11527	-0,683	GO:0050768	negative regulation of neurogenesis	-1,75271	0
GO:0045981	positive regulation of nucleotide metabolic process	-3,07991	-0,663	GO:1901215	negative regulation of neuron death	-1,31067	0
GO:1900544	positive regulation of purine nucleotide metabolic process	-3,07991	-0,663	GO:1903828	negative regulation of protein localization	-1,36289	0
GO:0071407	cellular response to organic cyclic compound	-3,07057	-0,661	GO:0015849	organic acid transport	-1,69513	0
GO:0046916	cellular transition metal ion homeostasis	-3,06142	-0,659	GO:0031346	positive regulation of cell projection organization	-1,34215	0
GO:1901699	cellular response to nitrogen compound	-3,02668	-0,631	GO:0043270	positive regulation of ion transport	-1,62271	0
GO:0098660	inorganic ion transmembrane transport	-3,00779	-0,619	GO:0010976	positive regulation of neuron projection development	-1,65935	0
GO:0002686	negative regulation of leukocyte migration	-2,9516	-0,57	GO:1903532	positive regulation of secretion by cell	-1,63277	0
GO:0030518	intracellular steroid hormone receptor signaling pathway	-2,92753	-0,559	GO:0030155	regulation of cell adhesion	-1,45767	0
GO:0010043	response to zinc ion	-2,92753	-0,559	GO:1902806	regulation of cell cycle G1/S phase transition	-2,00515	0
GO:0071320	cellular response to cAMP	-2,90394	-0,542	GO:0060284	regulation of cell development	-1,32699	0
GO:1901653	cellular response to peptide	-2,8884	-0,533	GO:0001558	regulation of cell growth	-1,60953	0
GO:0006939	smooth muscle contraction	-2,88081	-0,532	GO:0032535	regulation of cellular component size	-1,86782	0
GO:0046683	response to organophosphorus	-2,81349	-0,471	GO:0008016	regulation of heart contraction	-1,30562	0
GO:0055076	transition metal ion homeostasis	-2,69223	-0,356	GO:0046883	regulation of hormone secretion	-1,88007	0
GO:0046942	carboxylic acid transport	-2,65384	-0,323	GO:0050796	regulation of insulin secretion	-1,69718	0
GO:0014074	response to purine-containing compound	-2,6465	-0,322	GO:0042391	regulation of membrane potential	-1,58257	0
GO:0071363	cellular response to growth factor stimulus	-2,60643	-0,307	GO:0090276	regulation of peptide hormone secretion	-1,48992	0
GO:0030522	intracellular receptor signaling pathway	-2,61315	-0,307	GO:0002791	regulation of peptide secretion	-1,47151	0
GO:0120254	olefinic compound metabolic process	-2,60221	-0,307	GO:0051223	regulation of protein transport	-1,34544	0
GO:0062012	regulation of small molecule metabolic process	-2,61929	-0,307	GO:0051046	regulation of secretion	-1,99155	0
GO:0007584	response to nutrient	-2,60221	-0,307	GO:1903530	regulation of secretion by cell	-1,66068	0
GO:0045926	negative regulation of growth	-2,5518	-0,264	GO:0051384	response to glucocorticoid	-1,7609	0
GO:0062013	positive regulation of small molecule metabolic process	-2,54877	-0,264	GO:0032868	response to insulin	-2,04744	0
GO:0071560	cellular response to transforming growth factor beta stimulus	-2,52796	-0,254	GO:0010038	response to metal ion	-1,30818	0
GO:0043401	steroid hormone mediated signaling pathway	-2,52806	-0,254	GO:0009410	response to xenobiotic stimulus	-1,68167	0
GO:0120035	regulation of plasma membrane bounded cell projection organization	-2,49246	-0,229	GO:0035725	sodium ion transmembrane transport	-1,91154	0
				GO:0006814	sodium ion transport	-1,47151	0
