# Supplementary material for: Downregulation of Enteroendocrine Genes Predicts Survival in Colon Cancer: A Bioinformatics-Based Analysis
Source: Int J Mol Sci. 2025 Nov 18;26(22):11127. doi: 10.3390/ijms262211127 (PMC12652218; doi:10.3390/ijms262211127)
Supplement: Supplementary file 1 [file ijms-26-11127-s001.zip › Supplementary/Manuscript_Supplementary_Figures_Legends_IJMS_v2.docx]

**Supplementary Figures legends**

**Figure S1.** Differential expression analysis of microarray data from experimental and human colorectal cancer tissue. **A, B.** Principal Component Analysis (PCA) plots of public microarray datasets from mouse GSE31106 **(A)** and GSE64658 **(B)** tissues, comparing normal colon (blue) and colitis-associated colorectal cancer (CAC) (red). Sample sizes are: AOM_DSS $n=6$ and Control $n=3$ **(A)**; $n=6$ per group **(B). C, D.** PCA plots of public microarray datasets from human GSE18105 **(C)** and GSE110225 **(D)** tissues, comparing normal colon (blue) and colorectal cancer (CRC) (red). $n=17$ per group. **E–H.** Volcano plots displaying differential gene expression comparing murine (GSE31106 and GSE64658) **(E, F)** and human (GSE18105 and GSE110225) **(G, H)** CRC tissue with adjacent normal tissue. Genes down and upregulated in cancer tissue are highlighted in blue and red, respectively. The analysis was performed using the limma package with cut-offs of False Discovery Rate ($\text{FDR}$) $<0.05$ (Benjamini–Hochberg method) and $\log_{2} FC$ (Fold Change) $<-1$ and $>1$.

**Figure S2.** Differential expression analysis of microarray data from experimental APC^min^ mice and human colitis-associated colorectal cancer. **A.** PCA plot of the public microarray dataset from APC-deficient mouse GSE107139, comparing normal colon (blue) and colorectal cancer (CRC) (red). $n=4$ per group. **B.** PCA plot of the public microarray dataset from human colitis-associated colorectal cancer (CAC) tissue GSE37283, comparing normal colon (blue) and CRC (red). CAC $n=11$ and Control $n=5$. **C.** Bubble-Based Heatmap showing differential expression between tumor and normal samples of intestinal epithelial cell (IECs) genes from APC^min^ mice (circle, GSE107139) and human CAC (square, GSE37283). The y-axis lists genes selected as specific markers of secretory IECs, along with their transcriptional regulators, as previously described in the literature. The x-axis represents the $-\log_{10} \left( \text{adj.p} \right)$. The vertical dotted line indicates the cut-off for $\text{FDR}<0.05$. The color intensity denotes the direction and level of the difference $\log_{2} FC$ between tumor and normal tissue, while the size of the bubble is related to the level of significance of the $\text{FDR}$ $-\log_{10} \left( \text{p.adj} \right)$. Generated using R (version 4.x; R Core Team, 2024) with the ggplot2, readr, dplyr, and scales packages.

**Figure S3.** Downregulation of enteroendocrine cell proteins and upregulation of Paneth cell proteins in human Colon Adenocarcinoma (COAD) tissue at the protein level. **A.** Bar graph showing the percentage of patients positive for Immunohistochemistry (IHC) staining of IEC-related proteins in tumor (red) and normal tissue (blue). The y-axis represents the percentage of positive samples for a given specific IEC marker. The x-axis represents protein markers for enteroendocrine cells (blue), goblet cells (green), Tuft cells (yellow), and Paneth cells (red). Note that more than one antibody could be used for each sample. IHC analysis performed using the Protein Atlas. **B, C.** Representative IHC images from Protein Atlas of COAD tissue. **B.** IHC marker for GCG protein in normal colon (left) and COAD tissue (right). **C.** IHC marker for DEFA1 protein in normal colon (left) and COAD tissue (right).

**Figure S4.** STRING interaction network of upregulated genes in human colorectal cancer (CRC) tissue. STRING analysis of common upregulated genes obtained from Venn Diagrams in human CRC using the minimum required interaction score with high confidence. Analysis performed using the string-db platform.

**Figure S5.** RNAseq analysis from The Cancer Genome Atlas (TCGA) platform showing the top 25 upregulated genes in Colon Adenocarcinoma (COAD) tumors. The top 25 upregulated genes in human COAD tissue from the TCGA RNAseq database. The color range reflects the $\log_{2}$-normalized Transcript Per Million (${log}_{2}$- $\text{TPM}$) values comparing normal and colorectal cancer tissue. The y-axis represents the Top-25 genes (symbol), while the x-axis represents the groups (normal and tumor). Analysis performed using the UALCAN platform.

**Figure S6.** Differential expression of intestinal epithelial cell (IECs) genes across cancer stages in human Colon Adenocarcinoma (COAD). **A–X.** RNAseq dataset from the TCGA COAD database. Differential expression analysis by Transcripts Per Million ($\text{TPM}$) of IECs markers across different cancer stages. Stages I, II, III, and IV are based on the American Cancer Society classification. Analyses performed through the Oncodb platform.
