# Supplementary figures and images for: Downregulation of Enteroendocrine Genes Predicts Survival in Colon Cancer: A Bioinformatics-Based Analysis
Source: Int J Mol Sci. 2025 Nov 18;26(22):11127. doi: 10.3390/ijms262211127 (PMC12652218; doi:10.3390/ijms262211127)

Fig. S1

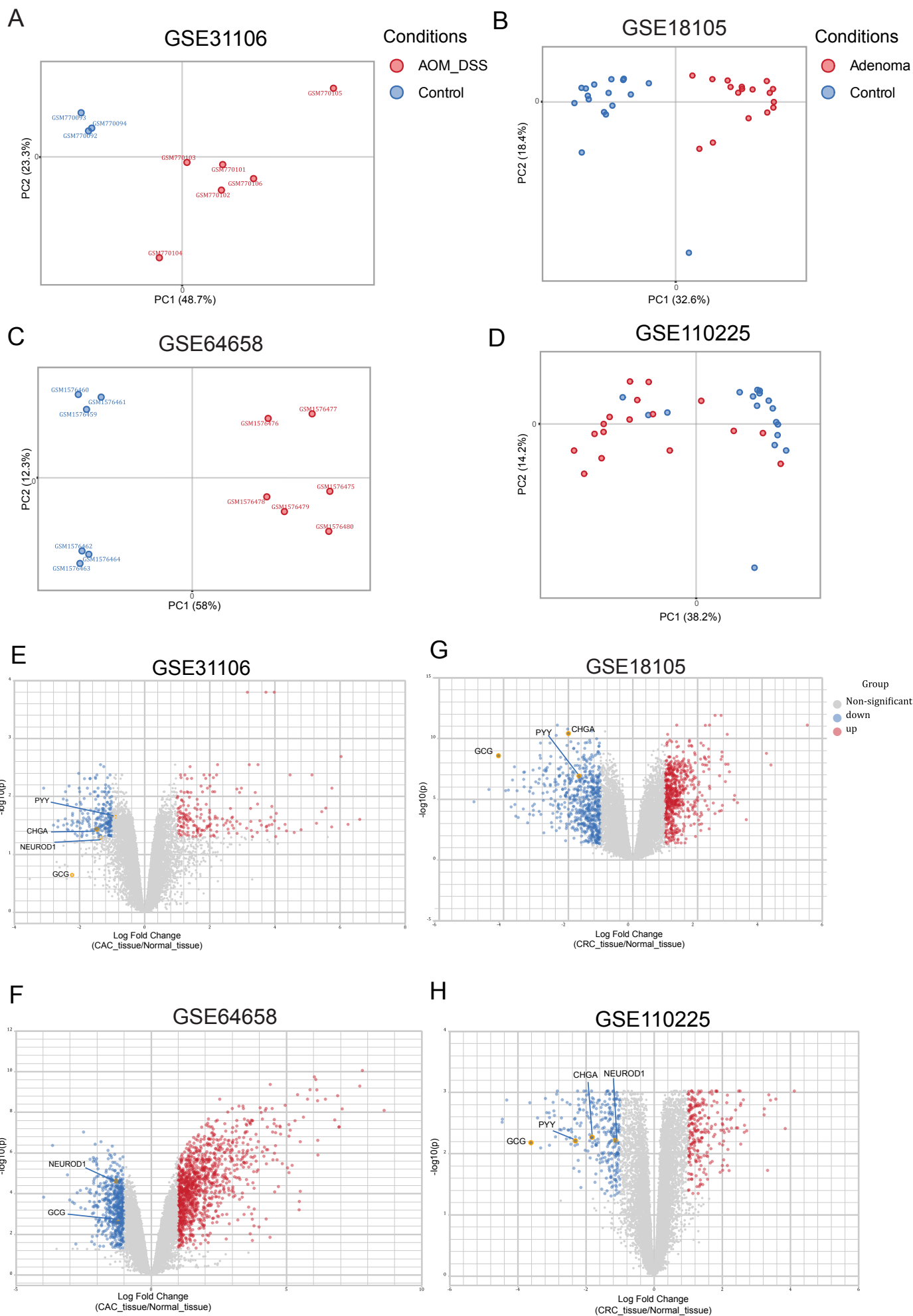

Fig. S2

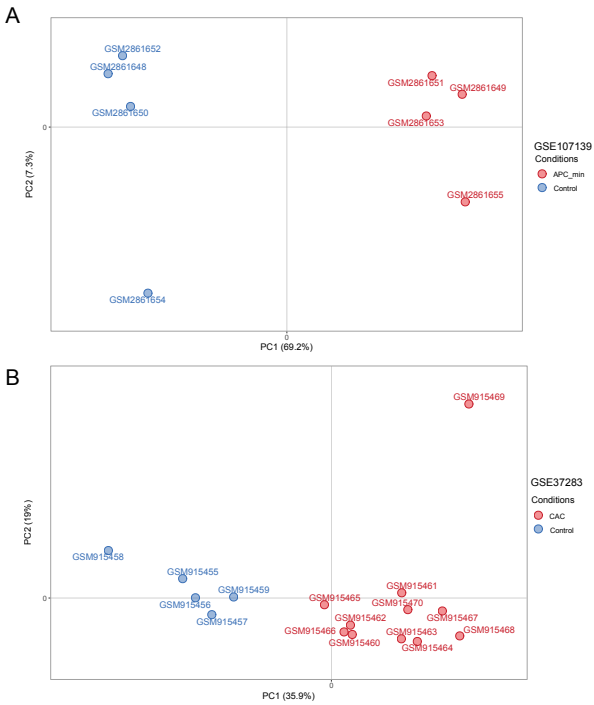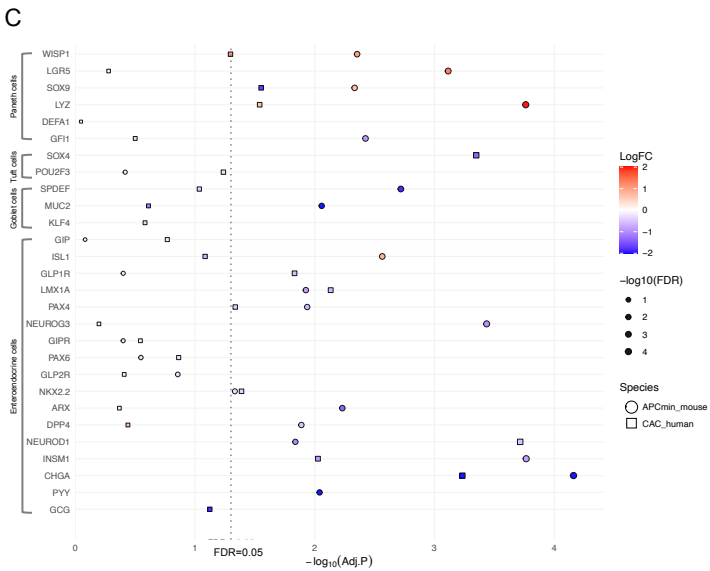

Fig. S3

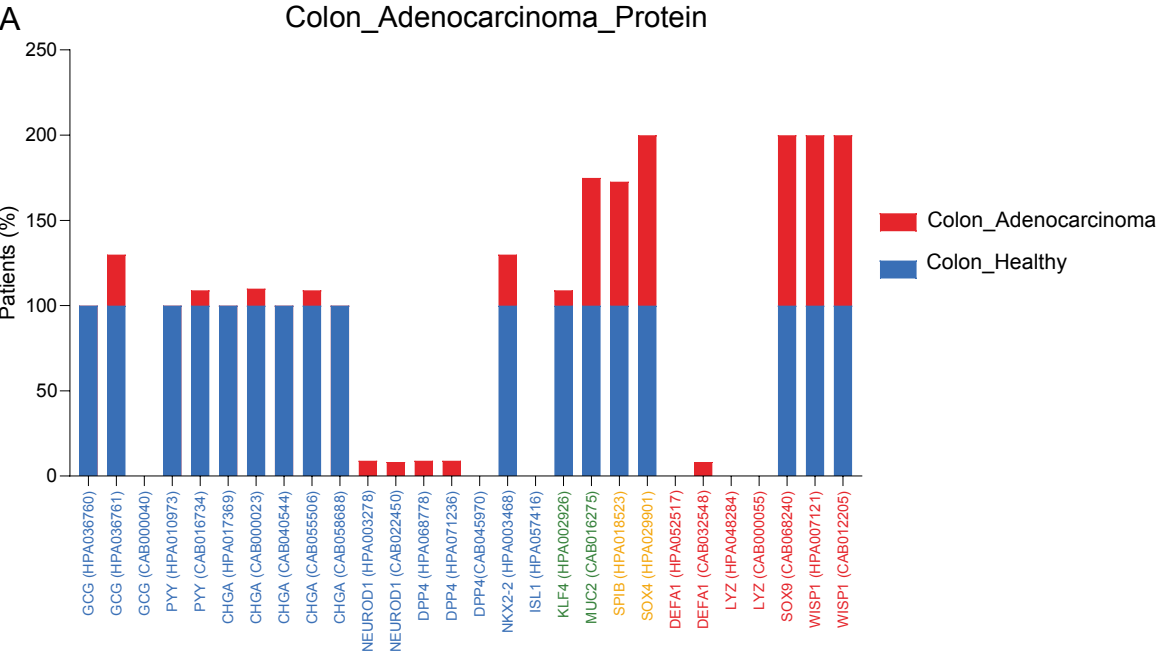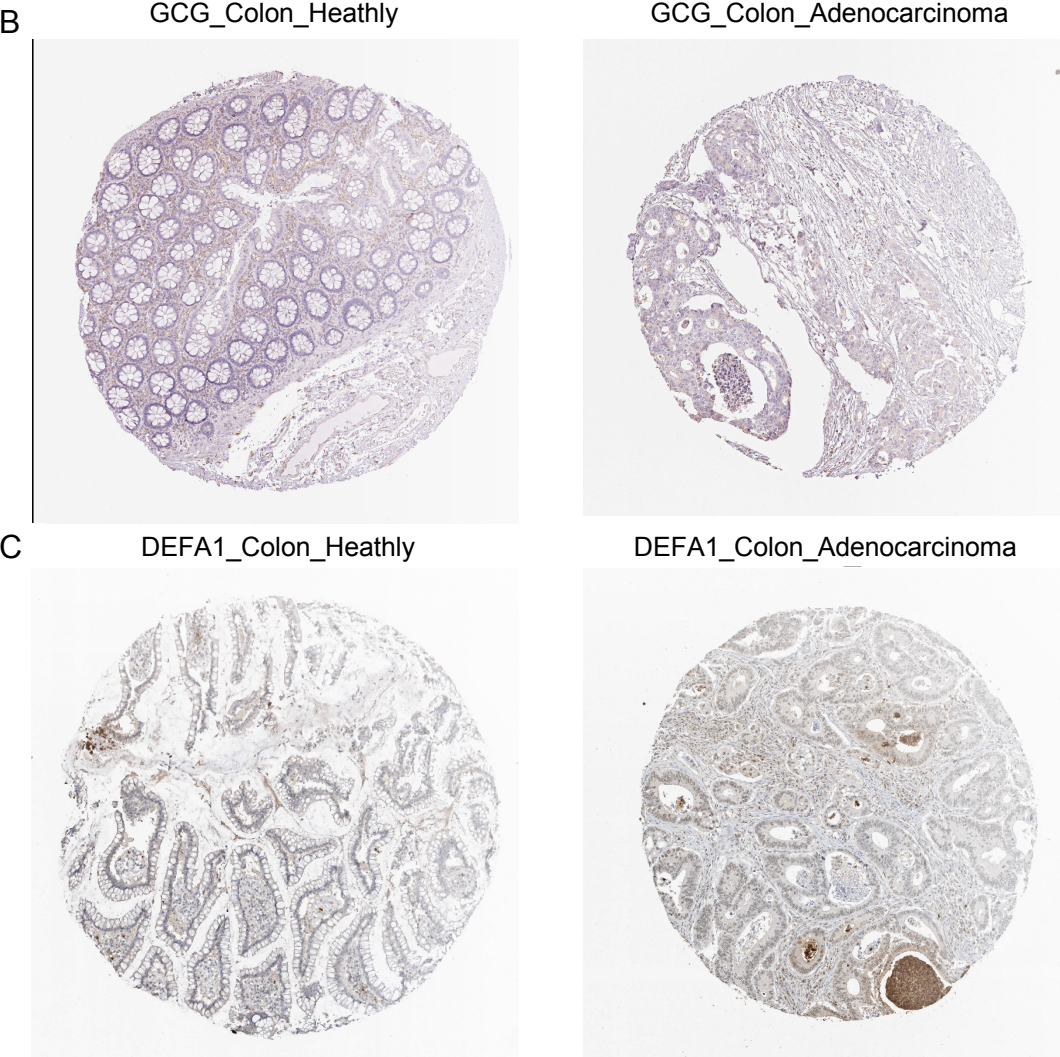

Fig. S4

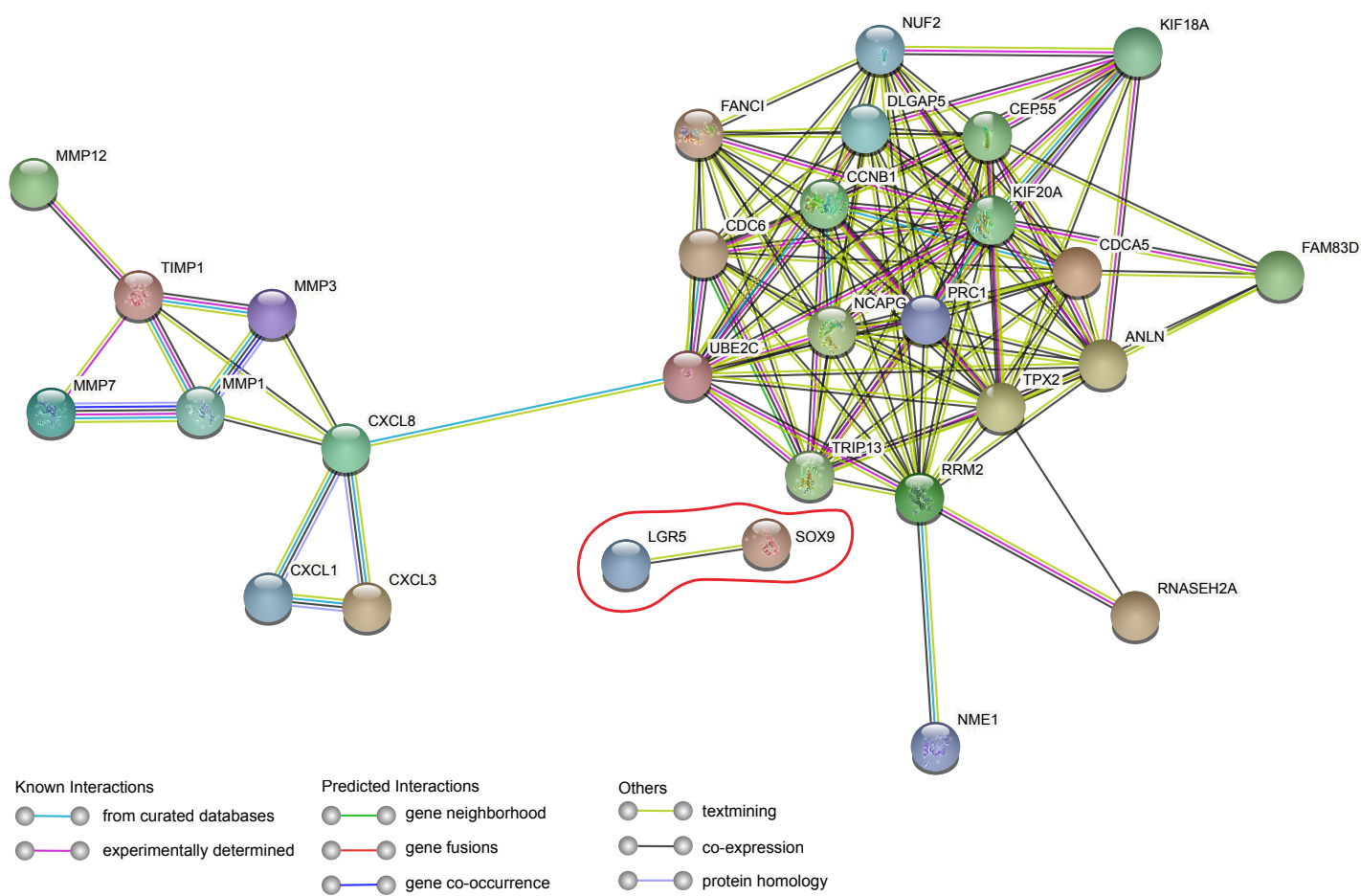

Fig. S5

Top 25 downregulated genes in  
Colon Adenocarcinoma (COAD)

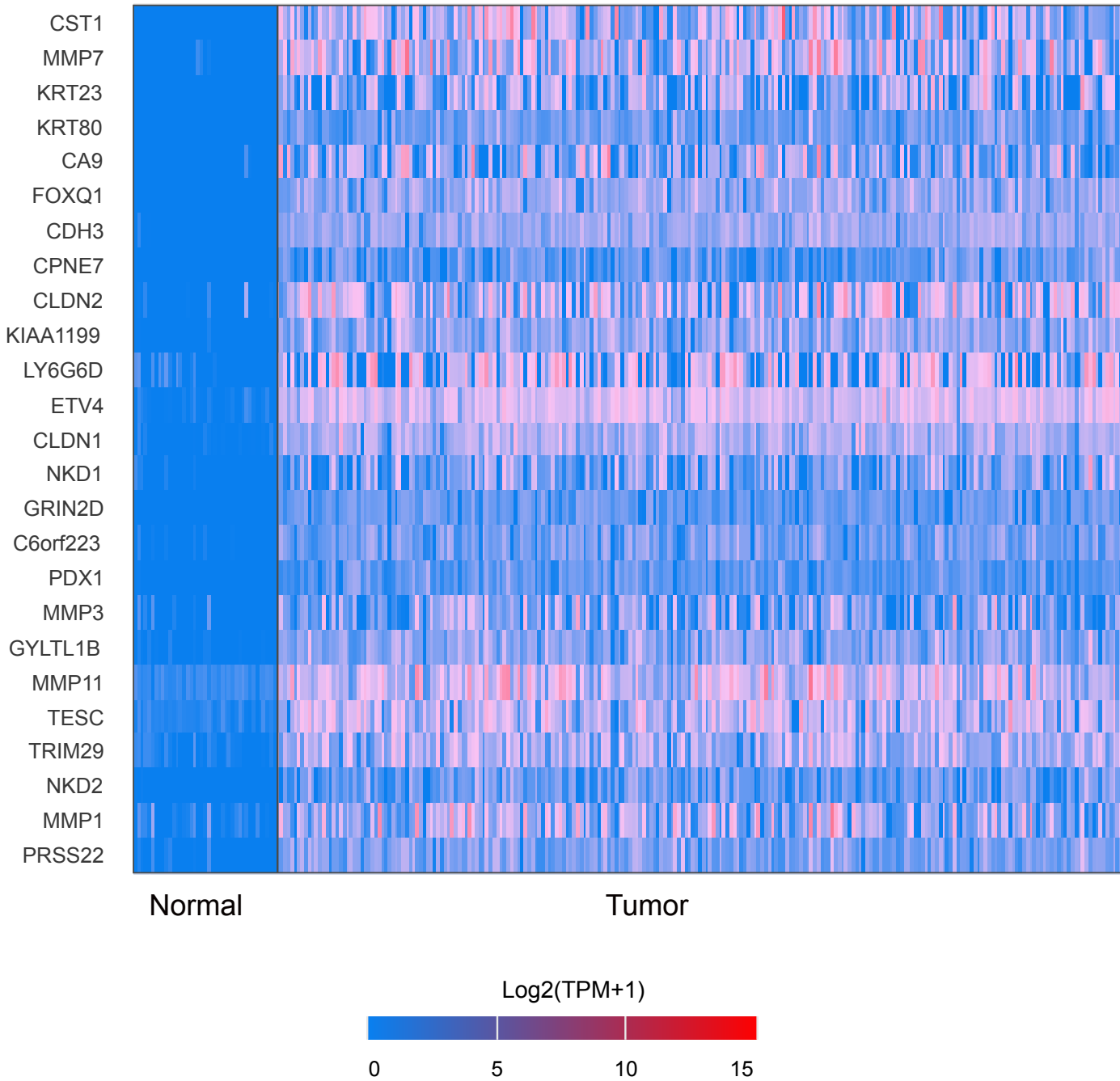

Fig. S6

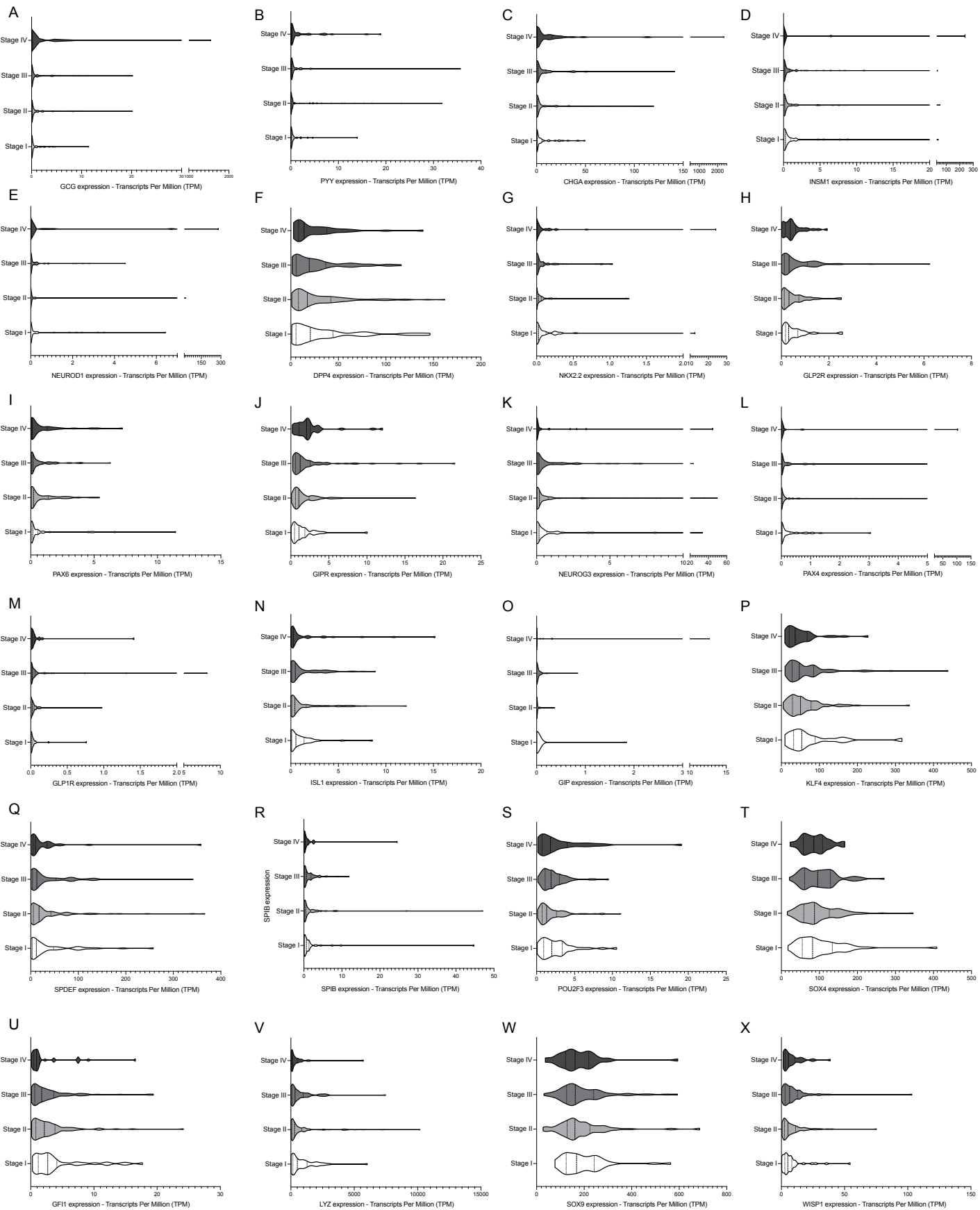

Supplement: Supplementary file 1 [file ijms-26-11127-s001.zip › Supplementary/Supplementary_Figures_IJMS.pdf]
